# Origin‐Dependence of Dipole Moments of Charged Proteins: Theoretical Foundations and Implications, Revisited

**DOI:** 10.1002/jcc.70207

**Published:** 2025-09-25

**Authors:** Islam K. Matar, Chérif F. Matta

**Affiliations:** ^1^ Department of Chemistry Saint Mary's University Halifax Nova Scotia Canada B3H 3C3; ^2^ Department of Chemistry and Physics Mount Saint Vincent University Halifax Nova Scotia Canada B3M 2J6

**Keywords:** ATP synthase, charged proteins, chemical education, coordinate origin dependence, dipole moment, macromolecular electrostatics, macromolecular modeling, macromolecular size effects, quantum mechanical Dirac observables, radius of gyration of biomolecules

## Abstract

Electric dipole moments are widely employed in structural biology and computational chemistry as global descriptors of macromolecular charge distribution, contributing to the understanding of protein interactions, solvation, and orientation in external fields. However, for systems bearing a nonzero net charge, the dipole moment becomes explicitly dependent on the choice of coordinates origin, a consequence grounded in classical electrostatics and sometimes overlooked in structural analyses. This origin‐dependence is particularly relevant in biological systems, as proteins are typically charged at physiological pH which differs from their isoelectric points (pI's). Moreover, coordinate manipulations such as centering and alignment are routinely performed during molecular dynamics simulations, docking, and structural comparisons, potentially altering the calculated dipole moment of charged systems. This study reviews the theory of the changes in the dipole moment of charged macromolecules accompanying displacements of the origin of the coordinates system. The theory is illustrated by numerical examples on representative proteins. Using the classical expression ${\vec{\mu }}^{\prime }=\vec{\mu }‐Q\vec{a}$, we demonstrate that displacements of the order of a protein's radius of gyration or larger can induce dipoles several hundreds to thousands of debyes. We examine this effect across a range of proteins with varying sizes and identify trends correlating the extent of origin‐induced changes with molecular size. These examples highlight the need for standardization in defining coordinate systems in dipole‐related analyses. The quantum mechanical status of the dipole moment operator is discussed clarifying that only neutral systems satisfy Dirac's criteria for a true “observable”. Altogether, theory, numerical benchmarks, practical guidelines, and pedagogical insights are presented for reliably calculating and interpreting dipole moments of charged biological macromolecules.

## Graphical abstract

The dipole moment of a charged protein (ATP synthase (with Q = +18 *e*)) is made to annul or even to reverse its sign by simple coordinate transformations, emphasizing the non‐physical nature of the dipole moment of a charged protein.

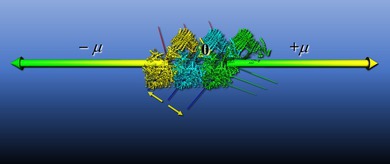

## 1 Introduction

It is fitting to dedicate this paper to Professor Shridar R. Gadre, a pioneer of molecular electrostatics among several others of his seminal contributions to theoretical chemistry [1, 2, 3, 4, 5, 6, 7, 8, 9, 10, 11]. Characterizing the electrostatic properties of biological macromolecules, such as proteins and nucleic acids, is crucial for understanding their molecular interactions and functions [6, 12, 13, 14, 15, 16, 17, 18, 19, 20, 21, 22, 23, 24]. A key property in this context is the electric dipole moment, which describes the spatial separation of the centers of positive and negative charges within a molecule. Before we proceed further, we remind the reader of the different types of molecular dipole moments that can exist in a molecule. What we are concerned with here is the “*permanent dipole*”, that is, the dipole moment arising from the asymmetric distribution of nuclear and electronic charges in a molecule's ground state which persists in the absence of external fields. An example is the permanent dipole moment of a water molecule due to its bent geometry and the difference in the electronegativity of oxygen and hydrogen atoms. This is to be distinguished from “*induced dipole*” which is a temporary dipole moment induced when an external electric field distorts its electronic cloud, which has a magnitude proportional to the polarizability and the intensity of the applied electric field. Finally, we are also not concerned here with the “*transition dipole*” which is the quantum mechanical expectation value of the electric dipole associated with an electronic or vibrational transition between two states. The latter governs the strength of light absorption or emission.

The dipole moment governs, at least in part, how molecules interact with external electric fields and with other molecules and ions. Dipole moments can influence the orientations of membrane proteins [12, 24], DNA‐binding propensities [25], pH‐induced conformational changes, and electrostatic complementarity in protein–ligand complexes [2, 4, 6, 12, 13, 14, 15, 16, 17, 18, 19, 20, 21, 22, 23, 24]. Meanwhile, proteins and nucleic acids often carry net electrical charges and exhibit significant dipolar polarization associated with their complex charge distributions. Such net charges render the dipole moment ambiguous which is the primary focus of this article.

The dipole moment $\vec{\mu }$ is defined for a system of point charges {*q*_*i*_} located at positions {$\vec{r_{i}}$} relative to a chosen origin. In this paper we are concerned with the permanent molecular dipole of the ground electronic state (not induced or transition dipole moments). The dipole moment has units of charge multiplied by length. For context, the gas‐phase dipole moment of H_2_O is 1.855 D as measured from microwave spectroscopy [26]. In condensed phases e.g., liquid water, the effective dipole moment is higher (typically estimated between 2.6 and 3.1 D) due to polarization and intermolecular interactions. In molecular science, the debye (D) is commonly used, where 1 D ≈3.33564 × 10^−30^ C·m. Theoretical studies often use atomic units (a.u.) in which 1 a.u. = *ea*_0_ ≈8.478 × 10^−20^ C.m ≈2.5417 D, where *a*_0_ is the Bohr radius. There are two sign conventions for dipole direction: the “*physicist‘s convention*” from (−) to (+) (lowest energy configuration when parallel to an external electrostatic field) and the opposite “*chemist‘s convention*” (from (+) to (−)) emphasizing electronic charge flow within molecules, e.g., due to bond polarization [27, 28, 29, 30]. Both conventions are, of course, equally valid. In this manuscript, we adopt the physicist‘s convention throughout and recommend that authors clearly specify the chosen convention when reporting dipole vectors to avoid ambiguity.

The dipole moment of a neutral molecule with an inherently asymmetric charge distribution is an experimentally measurable quantity [31]. Flygare [32] describes the theory of gas‐phase rotational microwave spectroscopy for measuring dipole moments of neutral molecules with a permanent dipole moment that are able to absorb microwave radiation in rotational transitions (see also the classic book: Ref. [33]). The Stark effect [33, 34, 35], that is, the shifts in the molecular energy levels induced by an external electric field, is proportional to the dipole moment. An analysis of Stark splittings in high‐resolution spectra yields dipole moment magnitudes and, in symmetric top molecules, the moments' directions [31]. Measurements in solution are more approximate and model‐dependent [36, 37]. Flygare does not address the dipole moment of charged species. Such moments are, as we emphasize in this article, ambiguous or ill‐defined without additional constraints or conventions.

The dipole term is the second term in the standard multipole expansion of the electrostatic potential or of the electrostatic field of an arbitrary set of charges as is well‐known [6, 38, 39]. The electrostatic potential $\Phi (\vec{r})$ at a field point $\vec{r}$ due to a localized charge distribution $\rho ({\vec{r}}^{\prime })$ is given in standard notation by:
(1)

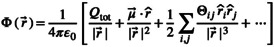

where $Q_{\mathrm{tot}}$ is the monopole (net) charge, $\vec{\mu }$ is the dipole moment vector, $\Theta_{\mathrm{ij}}$ is the traceless quadrupole moment tensor, $\hat{r}=\vec{r}⁄|\vec{r}|$ is the unit vector in the direction of $\vec{r}$, ${\hat{r}}_{i}{\hat{r}}_{j}$ refers to the dyadic (tensor) product of the unit vector with itself, i.e. $\hat{r}⊗\hat{r}$, and where the second term in the expansion is the contribution of the dipole to the electrostatic potential. Higher‐order terms (e.g., octupole, hexadecapole) fall off as $|\vec{r}{|}^{-n}$ with increasing power n≥4. Equation (1) holds for *r* larger than the radius of the smallest sphere enclosing the charge distribution; truncation after the 1/*r* ^3^ term incurs an O(*r* ^−4^) error. Taking the negative of the gradient of the expression for the potential yields the corresponding expression for the field [6]:
(2)

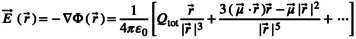

where the second term is the dipole contribution to the electrostatic field:

(3) $${\vec{E}}_{\mathrm{dip}}(\vec{r})=\frac{1}{4\pi \varepsilon_{0}}\left[\frac{3(\vec{\mu }\cdot \hat{r})\hat{r}-\vec{\mu }}{|\vec{r}{|}^{3}}\right]$$

which falls off as $|\vec{r}{|}^{-3}$ (quadrupole and higher‐order terms decay even faster). For charged systems the dipole moment term depends on the choice of coordinate origin (*vide infra*). Therefore, unlike the monopole term, the dipole contribution *is not an intrinsic property of the molecule in a charged system*, it depends on the spatial reference frame.

This article aims to re‐examine the theoretical foundations and structural consequences of origin‐dependent dipole moments in charged proteins. In computational biochemistry, bioinformatics, and molecular biophysics, various conventions have been adopted to address this origin dependence; yet none appears to have been universally adapted especially for large flexible macromolecules with complex and dynamic charge distributions [15].

## 2 The Origin‐Dependence of the Dipole Moment of Charged Macromolecules

An important consideration in calculating dipole and higher‐order multipole moments is their possible dependence on the choice of coordinate system and its origin. For systems with a net charge (*Q* ≠ 0), all multipoles in Equations (1) and (2) beyond the monopole are origin‐dependent. Under a translation of the origin by $\vec{a}$ (so that ${\vec{r_{i}}}^{\prime }=\vec{r_{i}}-\vec{a}$), the rank‐*l* multipole acquires terms up to order *Qa* ^*l*^ (e.g., dipole *l*=1: *Qa⃗*; quadrupole *l*=2: *Q* $\vec{a}⊗\vec{a}$). In general, the scaling of terms goes as *Q a* ^*n*−1^ with *n* = 2 for dipole, *n* = 3 for quadrupole, etc. Of course, there is a precise mathematical cancellation of this non‐physical origin dependence if the entire expansion is included to reconstruct the electrostatic potential or its derived field. While individual multipole moment terms in expansions (1) and (2) for a charged system are origin‐dependent, the full electrostatic potential they generate is not, *provided the complete multipole expansion is used consistently around a given origin*. Here, however, we are interested in only one of the multipoles that are often discussed in isolation and without reference to the full expansion, that is, the molecular dipole moment. If the molecule is charged, this term on its own is non‐physical since it is origin‐dependent. Great caution must, hence, be exercised in any discussion relating to the dipole moment of a macromolecule which is likely to be charged. Understanding the reasons for this origin dependence and its possible dependence on, say, molecular size, charge, or magnitude of origin displacement is essential for accurate modeling of electrostatic interactions in biological macromolecules.

In the following discussion, and without loss of generality, we will assume that the charge density of a protein can be approximated by a set of effective atomic partial charges centered at the positions of the nuclei of the atoms composing the protein of interest. An example of such charges that can be used to approximate the charge density of a gigantic protein is those used in molecular mechanics force fields [40] such as CHARMM [41, 42] or AMBER charges [43, 44, 45, 46, 47].

Let $\vec{r_{i}}$ be the position vector of the *i* ^th^ atom (with *N* being the total number of atoms in the structure) in a coordinate system *S* and let its associated partial charge be *q*_*i*_. The dipole moment in *S* is, then:

(4) $$\vec{\mu }=\sum_{i}^{N}q_{i}\vec{r_{i}}$$

For electrically‐neutral systems, this quantity is independent of the choice of origin. However, for systems with nonzero net charge $Q=\sum_{i}q_{i}\neq 0$, the dipole moment becomes origin‐dependent, since, if the origin is translated by $\vec{a}$, such that ${\vec{r_{i}}}^{\prime }=\vec{r_{i}}-\vec{a}$, the transformed dipole becomes [48]:

(5) $$\vec{\mu^{\prime }}=\sum_{i}^{N}q_{i}\left(\vec{r_{i}}-\vec{a}\right)=\vec{\mu }-Q\vec{a}$$

where the change in the dipole moment is linearly proportional to the net charge *Q* and the displacement $\vec{a}$. In magnitudes we have:

(6) $$\left|\Delta \vec{\mu }|=\left|Q\right|.|\vec{a}\right|$$

For a discussion about the meaning and implementation of equation (6) see the Appendix. Thus, even a modest shift in origin (e.g., by $\vec{a}$ (Å) = (+20, +20, +20), $\left|\vec{a}\right|$ = 34.64 Å) can induce changes of hundreds of debyes in a highly charged system (Table 1). The table shows that the selected proteins have a wide range of sizes (small, intermediate, and large) as can be judged from their molecular weight and radii of gyration. The table gives the net charge at neutral pH and the origin‐dependent dipole moment at that pH in addition to the origin‐independent dipole moment calculated for the neutral protein each at its isoelectric point (i.e., at pH = pI). We make a few general observations related to this table. The first is that the % change in the magnitude of the dipole moment is generally larger the greater the magnitude of the total charge (but this is *not* a simple proportionality relation due to the different angles between the displacement vector applied to all structures and the dipole moments of the different proteins. Equation (6) can be deceptive on the surface, as discussed in the Appendix). The second observation is that the *direction* of this change (increasing/decreasing) is unpredictable from the given information (see the last entry where the transformed dipole moment magnitude is lower than the un‐transformed system, opposing the trend for the other four entries). Finally, the magnitude of the % change can be enormous and for a highly charged system such as the histone octamer (with a net charge of +73 |*e*|) the magnitude of the transformed dipole moment is 64 times larger than the original dipole for a displacement of ≈35 Å. The effect of the displacement of the origin can, hence, be considerable.

**TABLE 1:** Origin‐induced dipole moment changes in some typical charged proteins (with their PDB codes) along with key properties including the molecular weight (MW), radius of gyration (*R*_*g*_), net (total) charge (*Q*), dipole moment magnitude ($\left|\vec{\mu }\right|$) in the original (centroid) coordinate system *S* (with the origin *O* = (0, 0, 0)) at neutral pH = 7.0, the magnitude of the transformed dipole moment ($\left|\vec{\mu^{\prime }}\right|$) that accompany the change to a coordinate system *S′* with axes parallel to *S* but with the origin *O′* displaced by $\vec{a}$ (Å) = (+20, +20, +20), $\left|\vec{a}\right|$ = 34.64 Å, and the % change in the magnitude of the dipole moment. Included in the table are also the magnitudes of the dipole moments of the electrically‐neutral forms of these proteins ($\left|{\vec{\mu }}_{\mathrm{pI}}\right|$), each determined at the protein's isoelectric point (pH = pI) which are unaffected by any coordinates changes. For details about the dipole calculations after the coordinate transformation see the Appendix.

Protein	PDB#	MW (kDa)	*R*_*g*_ (Å)	*Q* (*e*)	pI	$\left|{\vec{\mu }}_{\mathrm{pI}}\right|$ (D)	$\left|\vec{\mu }\right|$ (D)	$\left|\vec{\mu^{\prime }}\right|$ (D)	Δ $\left|\vec{\mu }\right|$ (%)
Lysozyme	1LYZ	14.3	14.6	+8	11.6	132	113	1269	1023.0%
Myoglobin	1MBN	17.2	15.5	+2	9.4	157	288	469	62.9%
Histone H1.0	7DBP	8.4	12.7	+10	10.6	242	140	1562	1015.7%
Histone Octamer	7DBP	85.2	28.9	+73	NA	NA	189	12288	6401.6%
RNAse A	7RSA	13.7	14.6	+4	10.4	388	479	205	−57.2%

**FIGURE 1:**
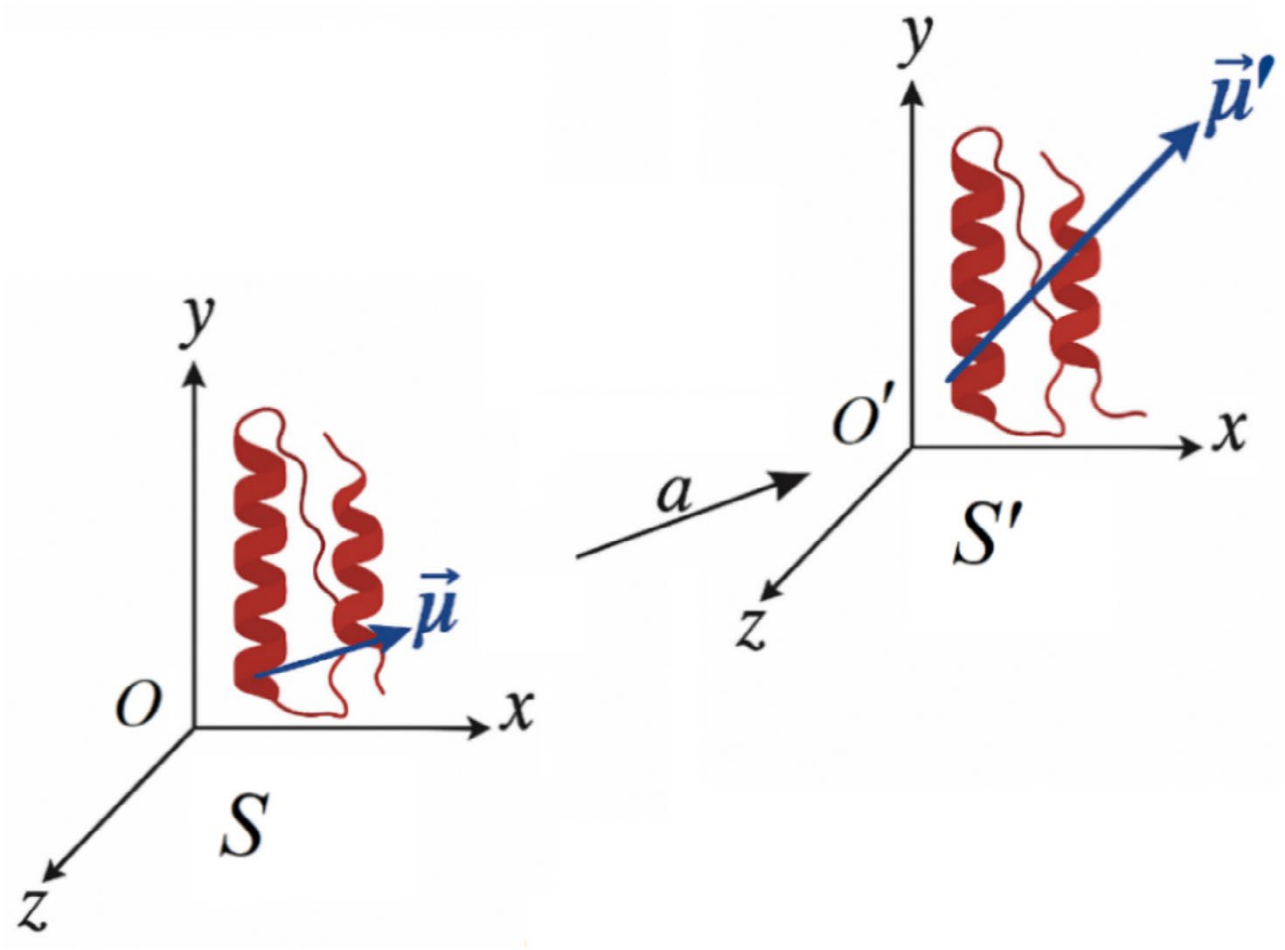
Diagram showing a shift of origin and the corresponding translation of the dipole moment vector of a charged peptide. The figure does not depict an actual peptide, instead, it conveys the relationship between a reference origin *O* in a coordinate system *S* and its (primed) counterpart after shifting the coordinate system by the displacement vector $\vec{a}$. The original and shifted dipole vectors are shown as blue arrows superposed on the hypothetical peptide. This diagram emphasizes that for charged systems, the dipole moment is not an intrinsic property but depends on the chosen coordinate system.

The origin‐dependence of the dipole moment of a charged peptide is graphically illustrated in Figure 1. The figure depicts the effect of shifting the coordinate origin on the dipole moment of a simple charged system. Although not based on a specific protein, the schematic shows a reference frame *S* centered at origin *O* = (0, 0, 0) and its displaced counterpart *O*′ offset by a vector $\vec{a}$. The associated dipole vectors $\vec{\mu }$ and $\vec{\mu^{\prime }}$ are depicted as blue arrows associated with a representation of a small charged peptide. We can take the dipole moment’s origin dependence to the extreme, that is, canceling the dipole moment of the charged system altogether. This can be achieved by re‐writing Equation (5) such that the modified dipole vanishes ($\vec{\mu^{\prime }}=0$):

(7) $$\vec{a}=\frac{\vec{\mu }}{Q}$$

**FIGURE 2:**
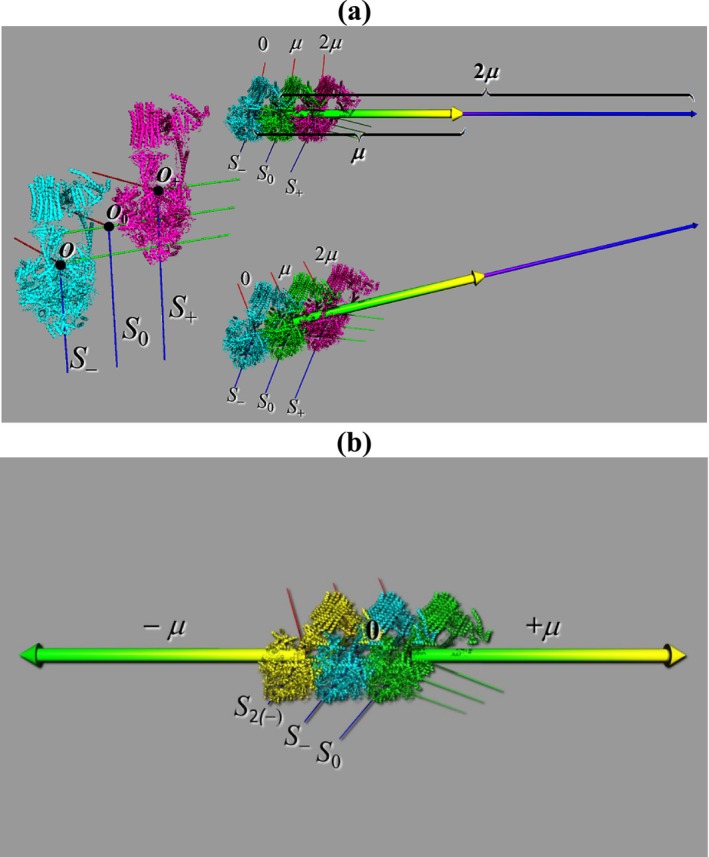
(a) Human ATP synthase (PDB ID: 8H9S, net charge +18 |e|) is shown in three coordinate systems: The centroid system *S*_0_ at *O*_0_ = (0, 0, 0), and two displaced systems *S*_−_ (cyan) and *S*_+_ (magenta) with origins ± (+14.935, −66.907, +42.900) Å, respectively. The dipole moment is 6990.8 D in *S*_0_, can be set to zero by coordinate translation to *S*_−_, and is doubled to 13981.6 D in *S*_+_. (b) The same structure is shown in *S*_0_ (green), *S*_−_ (cyan), and a further shifted system *S*_2(−)_ (yellow) with origin *O*_2(−)_ = (+29.870, −133.814, +85.800). The dipole in *S*_2(−)_ has the same magnitude but *reversed* direction relative to *S*_0_, illustrating that dipole magnitude and orientation of a charged molecule can be arbitrarily manipulated by origin shifts.

This result is illustrated Figure 2 where displacements are made to double, cancel, or even invert the dipole moment vector of a charged protein. Thus, human ATP synthase (PDB ID: 8H9S—with a net charge *Q* = +18 |*e*|) is displayed in the figure along with a vector representing its origin‐dependent dipole moment in several coordinate systems: The original frame *S*_0_ centers the structure such that the origin is placed at the average of the Cartesian coordinates of all the atoms (including the coordinates of the hydrogen atoms). We will call this origin the “centroid of the structure”. The centroid is given the designation *O*_0_ = (0, 0, 0) and, if clear from the context, as simply “*O*” without the subscript. In part (a) of Figure 2, *O*_0_ is moved to two symmetrically displaced frames (*S*_−_ and *S*_+_), which are centered, respectively at *O*_−_ = (+14.935, −66.907, +42.900) Å and *O*_+_ = (−14.935, +66.907, −42.900) Å, and the respective dipole moments re‐evaluated with PyMOL [49]. The calculated dipole moments with respect to the different coordinate systems exhibit dramatic differences. When ATP synthase is centered at *O*_−_, its dipole moment *is set to zero by coordinate translation* (∣*μ*_−_∣ = 0 D). In contrast, at the original frame of reference (*S*_0_) and its opposite‐shifted counterpart (*S*_+_), the dipole magnitudes are 6990.8 D and 13981.6 D, respectively. The colinear nature of the vectors and the doubling of the magnitude from coordinate systems *S*_0_ to *S*_+_ highlight that displacing the coordinate origin by a distance of the order of the molecular size can dramatically *amplify* or *completely suppress* the dipole moment of a charged protein. This serves as a demonstration of the coordinate origin dependence of the dipole moment in a charged macromolecule such as ATP synthase. Similar conclusions were reached from the empirical work of Ahmad and Sarai who realized that the dipole moment of a charged protein always vanishes if the origin coincides with the center of charge [25].

## 3 Scaling With Molecular Size?

The size of a mechanical system can be captured by various parameters. A simple but crude parameter is the difference between extreme coordinates of the system. The latter does not account for either the charge distribution or the atomic mass distribution and is purely geometric. In contrast, the radius of gyration describes how extended is, on average, the mass of the mechanical system. Meanwhile, in this work, we are more interested in the spreading of charge in space (and not how mechanical mass is spread). A parameter that characterizes molecular size in terms of the spread of its charge distribution is the *charge‐weighted radius of gyration* (*R*_*g*_), which provides a measure of the distribution of the molecular charge around the center of charge (CC):

(8) $$R_{g}\equiv \sqrt{\frac{\sum_{i}^{N}eq_{i}|\vec{r_{i}}-\vec{r_{\mathrm{CC}}}{|}^{2}}{\sum_{i}^{N}eq_{i}}}$$

or

(9) $$R_{g}\equiv \sqrt{\frac{1}{q_{\text{total}}}\sum_{i}^{N}q_{i}|\vec{r_{i}}-\vec{r_{\mathrm{CC}}}{|}^{2}}$$

where $\vec{r_{\mathrm{CC}}}$ is the center of charge of the charge distribution. Note that this is an electrical analogue of the mechanical “radius of gyration” whereby it is *not* weighted by the masses of the particles (*m*_*i*_) as in mechanics ($R_{g}=\sqrt{\frac{1}{M}\sum_{i}m_{i}|\vec{r_{i}}-\vec{r_{\mathrm{CM}}}{|}^{2}}$) but rather it is weighted by electric charges since the dipole moment and its origin dependence are properties of the charge distribution and not of the mass distribution.

For a neutral molecule, the charge‐weighted radius of gyration *R*_*g*_ is ill‐defined since, as can be seen from Equations (8) and (9), this would involve a division by zero. We will not bother in this work to define a “charge‐neutral” modified measure since for a charge neutral molecule, the dipole is unambiguously defined.

In proteins, the displacement vector $\vec{a}$ associated with different coordinate conventions is typically of the order of the radius of gyration $R_{g}$. If this is the case, then the induced origin‐dependent component of the dipole magnitude scales as:

(10) $$|\Delta \vec{\mu }|\approx \left|Q\right|.R_{g}$$

i.e., for a given total charge, the change in the dipole moment would be roughly proportional to the molecular size. This is exactly what we can subsume from the example given in Figure 2, whereby we can suppress or double the dipole moment vector by judicious displacements of roughly the order of magnitude of the ATP synthase’s molecular size. This underscores the importance of standardizing coordinate systems, particularly when comparing dipole magnitudes across proteins of differing sizes.

## 4 Numerical Examples

To assess the practical consequences of dipole origin‐dependence, we implemented a Python workflow that interfaces with PyMOL [49], beginning by loading atomic coordinates and assigned partial charges from a PQR file generated during pre‐treatment with the PDB2PQR module [50, 51] of the APBS solver/server [52, 53]. The structures are then re‐centered to a reference origin *O*_0_ = (0, 0, 0) (the centroid of the structure as explained above). The dipole moment is then computed in the *S*_0_ coordinate system in atomic units (a.u.) and in debyes (1e·Å=4.803D) using Equation (4). The computation is then repeated for the same structure shifted by an arbitrary displacement vector $\vec{a}$ (equation (5)).

As a numerical example, consider a hypothetical protein with net charge =+6e, shifted by $\vec{a}=\left(10,0,0\right)$ Å, that is 10 Å in the *x*‐direction. The change in the dipole moment due to $\vec{a}$ is:

(11) $$\Delta \mu_{x}=-Qa_{x}=-6e\cdot 10\;Å=-60\;e\cdot Å\approx -288.2\;D$$

For more realistic examples, Table 2 lists data related to eight proteins. The pH has been adjusted through a titration curve procedure (see Appendix and the accompanying Supporting Information (SI)—Figures S1–S8 and Tables S1 and S2) such that the first four have a net charge of *Q* = +10 |*e*| while the four others each has a net charge of *Q* = −12 |*e*|. Hence, unlike Table 1, here the pH was adjusted such that the total charge of every protein assumes a preset value, then we displaced the origin and re‐calculated the new dipole moment. The total charge of each protein is set to a common value by varying the pH followed by a uniform shift of origin of the same magnitude as the one performed to shift the origin in Table 1, i.e., the origin *O′* is displaced, again, by $\vec{a}$ (Å) such that *O′* = (+20, +20, +20) Å, $\left|\vec{a}\right|$ = 34.64 Å. A glance at Table 2 reveals that the magnitudes of the dipole moments of these proteins can change (increase) by *ca*. 170% (for cytochrome c peroxidase) to a staggering *ca*. 1600% for egg‐white lysozyme. Proteins with comparable net positive and negative charges (+10 to −12 |*e*|) exhibit similar scaling behaviors in the change of their dipole moments' magnitudes. Variations in the change in dipole moment magnitudes among proteins with the same nominal charge lysozyme (6LYZ) shows a 1549% increase, BPTI (4PTI) 683% increase, avidin (1AVD) 287% increase, and cytochrome c 609% increase ‐ despite all having 𝑄 = +10 |*e*| ‐ suggests that charge distribution, molecular geometry, and protein folding influence the baseline dipole moment $\vec{\mu }$, and hence, the shift‐induced amplification.

**TABLE 2:** Origin‐induced dipole moment changes in some representative charged proteins along with their molecular weight (MW), net (total) charge (*Q*) (set to +10 |*e*| and −12 |*e*| by adjusting the pH (see Appendix and SI)), dipole moment magnitude ($\left|\vec{\mu }\right|$) in the centroid coordinate system (*O* = (0,0,0)) and the magnitude of the transformed dipole moment ($\left|\vec{\mu^{\prime }}\right|$) in the new coordinate system *S′* with axes parallel to *S* but with the origin *O′* displaced by $\vec{a}$ (Å) such that *O′* = (+20, +20, +20), $\left|\vec{a}\right|$ = 34.64 Å.

Protein (source)	PDB code	*Q* (*e*)	MW (kDa)	Function (class)	$\left|\vec{\mu }\right|$ (D)	$\left|\vec{\mu^{\prime }}\right|$ (D)	Δ$\left|\vec{\mu }\right|$ (%)
Proteins bearing a net positive charge *Q* = +10 |*e*|							
Egg‐white lysozyme (chicken)	6LYZ	+10	14.3	Enzyme (glycosidase)	96	1583	1549%
Cytochrome *c* (horse heart)	1HRC	+10	11.7	Electron carrier (heme)	235	1667	609%
Avidin (monomer) (chicken egg)	1AVD	+10	13.7	Biotin‐binding (transport)	539	2083	287%
Pancreatic Trypsin Inhibitor (bovine BPTI)	4PTI	+10	6.5	Protease inhibitor	228	1784	683%
Proteins bearing a net negative charge *Q* = −12 |*e*|							
Plastocyanin (spinach)	1AG6	−12	10.5	Electron carrier (Cu protein)	303	2174	618%
Cytochrome *c* Peroxidase (yeast)	2CYP	−12	33.4	Enzyme (oxidoreductase)	758	2035	169%
β‐Lactoglobulin (cow milk)	3NPO	−12	18.3	Lipid‐binding (storage protein)	543	2469	355%
α‐Lactalbumin (bovine milk)	1HFY	−12	13.8	Ca‐binding enzyme (lysozyme homolog)	434	1745	302%

The large changes in dipole moments' magnitudes in Tables 1 and 2, call for caution when interpreting their biological relevance. In practice, dipole moments are often used to guide or interpret biological modeling tasks such as protein‐ligand docking, membrane alignment, or electrostatic complementarity scoring [40]. A wrong dipole moment magnitude or direction due to coordinate origin ambiguity can lead to misinterpretation of interaction strengths, incorrect alignment of dipole vectors with respect to electric fields across membranes (e.g., inner mitochondrial membrane or nerve membranes), or artificial enhancement/suppression of electrostatic match between macromolecules. For example, a membrane protein, e.g., ATP synthase, predicted to insert with a given orientation based on a large dipole may instead show the opposite behavior if the coordinate system used was misaligned. Similarly, docking algorithms that rely on global electrostatics could produce suboptimal poses if the dipole exaggerates or underestimates molecular polarity. Thus, origin‐induced dipole artifacts can introduce systematic (possibly serious) errors into biologically significant computational workflows. Careful origin specification and, where possible, use of coordinate‐invariant methods (e.g., neutral subdomains or constrained environments) are crucial for reliable biological interpretations.

## 5 Structural Databases

The RCSB Protein Data Bank (RCSB PDB) [54] structures often lack a standardized definition of the coordinate systems in which its various entries are expressed. Crystallographic protein structures are arbitrarily placed within the crystallographic unit cell. Without re‐centering, dipole computations across such entries can yield inconsistently biased results. Felder et al. [55] developed the Protein Dipole Moments Server (PDMS) [56] including a database (The DIPole database) providing pre‐computed dipole moments for thousands of proteins based on PDB structures. The dipole moments are calculated using atomic partial charges and are presented alongside such data as center of mass and orientation. This resource is useful for structural and electrostatic analyses, particularly for membrane‐protein interactions and docking studies. While the calculations assume vacuum conditions and fixed protonation states, the database remains useful for comparative protein electrostatics. Felder et al.'s PDMS calculates the dipole moment of proteins in a standardized and reproducible way (with the orientation given in the physicist's convention) by first applying the following preprocessing steps before computing the dipole vector:

1. Origin centering by translation to the geometric center (centroid). In other words, the average *x*, *y*, *z* coordinates over all heavy atoms (not including hydrogens) [57] in the structure are moved to the origin (0, 0, 0), i.e.,
(12) $${\vec{R}}_{\text{centroid}}=\frac{1}{N}\sum_{i=1}^{N}\vec{r_{i}}=\left(0,0,0\right)$$
  where $\left\{{\vec{r}}_{i}\right\}$ represent the set of atomic positions and *N* is the number of non‐hydrogen atoms in the protein structure. This re‐centering ensures consistency across structures from the Protein Data Bank (PDB), which generally have arbitrary coordinate origins.
2. After centering, the protein is aligned with its principal axes of inertia. This is achieved by computing the moment of inertia tensor of the atomic distribution followed by diagonalizing this tensor to find the eigenvectors (principal axes); and then rotating the protein so that its first, second, and third principal axes align with the *x*‐, *y*‐, and *z*‐axes, respectively. This “canonical orientation” eliminates the arbitrariness of the original coordinate system and facilitates consistent dipole direction comparisons across proteins.
3. Once the protein has been oriented as described above, each atom is then assigned a partial charge based on its chemical identity using standard force field‐based charges (e.g., from AMBER [43], CHARMM [41], etc.). Hydrogen atoms are inferred if absent using a heuristic or external tool (e.g., PDB2PQR) [50, 51, 52, 53].
4. The electric dipole moment vector is now computed from Equation (4); the result is reported in debye units (1 e·Å = 4.803 Debye).

Trajectories obtained from molecular dynamics (MD) simulations often include shifted or translated structures. Although the Protein Dipole Moments Server [56] centers and aligns structures in a consistent way, this does not eliminate the fundamental fact that the dipole moment of a system with nonzero net charge is origin‐dependent ($\vec{\mu^{\prime }}=\vec{\mu }-Q\vec{a}$). Thus, even after the standardized geometric centering and orientation described above, if two researchers use slightly different conventions for alignment or centroid definition (e.g., center of mass vs. centroid or vs. center of electric charge), the computed dipole can differ *significantly*, especially for highly charged or elongated systems. Other structural bioinformatics databases (e.g., PDB, AlphaFold DB, ModBase) do not enforce coordinate origin conventions. Researchers downloading structures from those sources and calculating dipoles locally (e.g., in PyMOL, GROMACS, AMBER) may obtain values that are not comparable.

To illustrate the situations when such ambiguity may become crucial, we mention proteins that bind nucleic acids (e.g., histones, Table 1) often exhibit large dipole moments aligned with their binding axis. In such highly charged proteins, this apparent dipole may reflect a particular coordinate choice rather than an intrinsic polarity; hence care must be exercised in relating this dipole to any biological property. Similarly, membrane proteins exhibit preferred orientations due to dipole interactions with the membrane electric field.

An elegant solution for dipole moment ambiguity of charged proteins has recently been proposed by Krawczuk et al. [58]. These authors presented a tool termed “GruPol”, a database‐driven approach using the Quantum Theory of Atoms in Molecules (QTAIM) [59, 60, 61] that can be used to compute coordinate‐invariant and pH‐dependent dipole moments of proteins with or without solvation effects and at any desired protonation state.

## 6 Is the Dipole Moment of a Charged Molecule Measurable? The Answer Is “No”

The origin‐dependent dipole moment of a charged molecule is *not a physical observable* unless an origin is fixed by convention or physical constraint. Alone, this dipole moment *does not* determine the orientation of the molecule in an external electric field.

The potential energy *U* of a system in a uniform electric field $\vec{E}$ is given by:

(13) $$U=-\vec{\mu }\cdot \vec{E}$$

For neutral molecules, this energy determines the torque $\vec{\tau }=\vec{\mu }\times \vec{E}$, which governs orientation. For charged systems, however, the total energy in an electric field also includes the monopole‐field interaction:

(14) $$U_{\text{total}}=-\vec{\mu }\cdot \vec{E}+Q\Phi$$

where Φ($\vec{r}$) is the electrostatic potential (in a non‐uniform field, higher‐order multipoles (e.g., quadrupole) and field gradients also contribute) [62]. If the origin is shifted by $\vec{a}$, the potential energy expression becomes:

(15) $$U_{\text{total}}=-{\vec{\mu }}^{\prime }\cdot \vec{E}+Q\Phi =-\left(\vec{\mu }-Q\vec{a}\right)\cdot \vec{E}+Q\Phi$$

which upon expansion yields:

(16) $$U_{\text{total}}=-\vec{\mu }\cdot \vec{E}+Q\vec{a}\cdot \vec{E}+Q\Phi$$

Thus, the change in energy from the shift is:

(17) $$\Delta U=Q\vec{a}\cdot \vec{E}$$

which appears to make the energy depend on the choice of origin, which can be viewed as problematic. However, for a conservative field such as the electrostatic field, the field is always the negative of the gradient of the potential, as is well known, and extracting this relation from Equation (2), we have:

(18) $$\vec{E}=-\nabla \Phi$$

If we now shift the origin, that shift in origin will also shift the electrostatic potential, and given that Φ is smooth (differentiable), we can expand $\Phi (\vec{r}+\vec{a})$ in a Taylor series around $\vec{r}$ and thus:

(19) $$\Phi^{\prime }(\vec{r})=\Phi (\vec{r}+\vec{a})$$

(20) $$=\Phi (\vec{r})+(\vec{a}\cdot \nabla )\Phi (\vec{r})+O(|\vec{a}{|}^{2})$$

(21) $$\approx \Phi (\vec{r})+\vec{a}\cdot \nabla \Phi$$

(22) $$=\Phi (\vec{r})-\vec{a}\cdot \vec{E}$$

where we have renamed the dummy argument $\vec{r}$′ as $\vec{r}$ for simplicity. We note in passing that the use of “≈”in Equation (21) after the Taylor expansion is for the general expression where we ignore higher order terms for a *non‐uniform* field. For a uniform field, however, Equation (13) is linear, the second derivatives vanish, and the Taylor series truncates exactly at first order. In this case, we may replace “≈” by “=” in Equation (21). Otherwise, the “≈” in Equation (21) denotes an invariance that holds to first order only in $\vec{a}$. Using this result and Equations (5), Equation (16) transforms to:

(23) $$U_{\text{total}}^{\prime }=-\vec{\mu }\cdot \vec{E}+Q\vec{a}\cdot \vec{E}+Q\Phi^{\prime }(\vec{r})$$

(24) $$=-\vec{\mu }\cdot \vec{E}+Q\vec{a}\cdot \vec{E}+Q\left[\Phi (\vec{r})-\vec{a}\cdot \vec{E}\right]$$

(25) $$=-\vec{\mu }\cdot \vec{E}+Q\Phi =U_{\text{total}}$$

exactly canceling the origin‐dependence from the dipole term.

While Equation (17) is correct, it does not represent a real physical change in energy, it arises from a *change in the mathematical representation* of the system due to the shift in coordinate origin. In other words, the mathematical *expression* for the energy has changed without changing the physical energy of the system since the apparent change $\Delta U=Q\vec{a}\cdot \vec{E}$ is canceled by the corresponding transformation of the scalar electrostatic potential as shown in Equations ((23), (24), (25)).

Jackson [38] and Stone [62], each discusses how the electric dipole moment depends on the origin for charged systems, and how the total electrostatic energy and physical observables remain invariant provided that the electric field derives from a scalar potential. These treatments rigorously derive the origin shift correction $\vec{\mu^{\prime }}\to \vec{\mu }-Q\vec{a}$ and shows the corresponding transformation of Φ.

In cases where physical constraints are imposed, such as when a charged protein is embedded in a membrane, anchored in a crystal lattice, or aligned by a uniform external field, *the coordinate origin is effectively fixed by the system's environment*. In these cases, the dipole moment, though formally origin‐dependent, can acquire physical meaning relative to the imposed frame. Moreover, analyzing the dipole moments of net‐electrically‐neutral domains (e.g., domains or subunits within a protein whose summed charge vanishes) provides an alternative path to defining origin‐independent, interpretable dipoles even within charged macromolecules. For neutral molecules, the dipole moment is both coordinate‐invariant and experimentally accessible and, hence, has an inherent physical meaning. For periodic systems, Ahrens‐Iwers et al. have recently shown analytically and computationally that the contribution of molecular dipoles to the local electrostatic potential in molecular dynamics (MD) simulations is not well‐defined *even for neutral molecules* [63]. This ambiguity arises from the arbitrary choice of a reference point within each molecule when defining its dipole. The authors conclude that decomposing the total electrostatic potential into dipole (and quadrupole) contributions can misrepresent the physics unless the reference choice is carefully controlled, or better, avoided entirely. In sum, discussions of dipole moments in macromolecular electrostatics should always specify which, if any, of these conditions apply.

## 7 Is the Dipole Moment of a Charged Molecule a Dirac Observable? The Answer Is “No”

Dirac defines a quantum mechanical “observable” (a quantity that is measurable in principle) as one that can be represented by a Hermitian (self‐adjoint, i.e., *Ô* = *Ô* ^†^) operator acting on the system's Hilbert space [64]. This ensures that the operator's eigenvalues (the possible measurement outcomes) are real numbers and that the operator has a well‐defined expectation value in any physical state. Dirac affirms that the expectation value (or “average value”) of an observable is given by the diagonal matrix element ⟨ψ|*Ô*|ψ⟩ when the operator *Ô* is Hermitian and the state |ψ⟩ is normalized [64].

The electric dipole moment of a set of discrete charges is defined by Equation (4). In quantum mechanics, the position vectors $\vec{r_{i}}$ become operators $\hat{\vec{r_{i}}}$, and the dipole moment operator becomes:

(26) $$\hat{\vec{\mu }}=\sum_{i}q_{i}\hat{\vec{r_{i}}}$$

This operator is Hermitian, since position operators $\hat{\vec{r_{i}}}$ are Hermitian and *q*_*i*_ are constants (charges). Thus, the dipole moment constitutes a “*quantum observable*” as it corresponds to a Hermitian operator and can have an expectation value for a given molecular state. For example, in a non‐degenerate electronic state of a molecule, one can compute $\left\langle \Psi |\hat{\vec{\mu }}|\Psi \right\rangle$ to obtain the molecule's permanent dipole moment vector in that state (where the sum in Equation (26) is applied to the point‐like positively charged nuclei and an integral replaces the sum for the (negatively) charged electron density). In general units, the quantum mechanical operator to compute the molecular dipole moment within the Born‐Oppenheimer approximation is:

(27) $$\hat{\vec{\mu }}=\frac{1}{4\pi \varepsilon_{0}}\left(\sum_{\alpha }Z_{\alpha }e\hat{\vec{R_{\alpha }}}\;-\sum_{i}e\hat{\vec{r_{i}}}\right)$$

which when expressed in atomic units (a.u.) becomes:

(28) $$\hat{\vec{\mu }}=\sum_{\alpha }Z_{\alpha }\hat{\vec{R_{\alpha }}}-\sum_{i}\hat{\vec{r_{i}}}$$

where *α* refers to the point‐like stationary nuclei and *i* to the electrons. The molecular dipole moment is computed as the expectation value of this operator in the usual fashion (in a.u.):

(29) $$\vec{\mu }=\langle \Psi |\hat{\vec{\mu }}|\Psi \rangle =\sum_{\alpha }Z_{\alpha }\vec{R_{\alpha }}-\int \vec{r}\;\rho (\vec{r})\;d^{3}r$$

where

(30) $$\rho (\vec{r})=N\sum_{\mathrm{spins}}\int |\Psi (\vec{r_{1}},\vec{r_{2}},...,\vec{r_{N}}){|}^{2}\;d^{3}\vec{r_{2}}...d^{3}\vec{r_{N}}$$

is the electron density, a continuous charge density replacing the sum over discrete electrons. In Equation (30), with a many‐electron wavefunction normalized to 1, the sum over all spin variables includes that of electron 1 (excluded from the integral over the spatial coordinates), which yields the definition of the spinless density, that is, $\rho =\rho_{\alpha }+\rho_{\beta }$.

The quantum mechanical analog of the origin‐dependence relation (Equation ((4), (5), (6), (7))) is:

(31) $$\hat{\vec{\mu^{\prime }}}=\hat{\vec{\mu }}-Q\;1⊗\vec{a}$$

where again $\vec{a}$ is the displacement vector of the coordinate origin, *Q* is the total molecular charge (nuclear plus electronic), and 1 is the identity operator. Taking the expectation value of this operator for normalized state ∣Ψ⟩, we obtain:

(32) $$\langle \hat{\vec{\mu^{\prime }}}\rangle =\langle \hat{\vec{\mu }}\rangle -Q\;\vec{a}$$

This expression is fully consistent with the classical result, confirming that the origin‐dependence of the dipole moment for charged systems arises identically in both quantum and classical formulations. Although the operator $\hat{\vec{\mu }}$ and its expectation value $\left\langle \hat{\vec{\mu }}\right\rangle$ are both origin‐dependent when *Q* ≠ 0, observable physical quantities, such as the energy of interaction between the charge distribution and an external electric field, remain origin‐invariant when all relevant contributions are properly accounted for. This dependence on the arbitrary choice of origin means that the expectation value of $\hat{\vec{\mu }}$ is not a physically invariant quantity for charged systems. In other words, the dipole moment changes based on the choice of coordinate system and, hence, it is not an intrinsic property of the quantum state. Therefore, although $\hat{\vec{\mu }}$ is still Hermitian, its expectation value is not physically well‐defined since it depends on the arbitrary choice of coordinate origin. *This violates Dirac's principle that observables must yield unique invariant values in a given state*. A charged molecule does not possess a unique invariant dipole moment in isolation since any value for $\left\langle \hat{\vec{\mu }}\right\rangle$ is “gauged” by where we set the origin. This emphasizes the importance of explicitly specifying the coordinate origin in dipole moment calculations of charged molecules, small or large, calculated classically or quantum mechanically, to avoid physically misleading interpretations or inconsistent comparisons. The dipole moment of a charged species is an *ill‐defined* or *gauge‐dependent* quantity. Bader expresses the contribution of a given atom‐in‐a‐molecule (AIM) to the molecular dipole moment as the sum of charge‐transfer terms between the atom in question and all its bonded neighbors and an (intra‐atomic) dipolar polarization term. Since most AIMs have net atomic charges, these “intra‐atomic dipoles” are generally origin‐dependent. Hence, Bader always refers this contribution to a coordinate system centered on the nucleus of the atom in question to avoid te gauge dependence of these dipoles [65, 66, 67].

In closing, and to offer an analogy from classical electromagnetic theory, consider how the electrostatic potential Φ is only defined up to an additive constant [38, 39]. Thus, Φ itself *is not an observable*, yet *differences* in potential are. For another analogy, the dipole moment of a charged system depends on the arbitrary coordinate origin much like the choice of a gauge in the expression of the electromagnetic vector potential $\vec{A}$ which has no effect on physical observables such as the magnetic field $\vec{B}$. This dependence on origin is analogous to the gauge dependence of $\vec{A}$ in the Aharonov‐Bohm (AB) effect [68, 69, 70]. In that phenomenon, electrons moving through a region where $\vec{B}$ is zero can still acquire a measurable phase‐shift in their wavefunction due to the presence of a non‐zero vector potential. This phase‐shift depends on the *difference between two paths* (i.e., *the interference pattern*) which is observable and not the absolute value of $\vec{A}$ itself which is not observable. Similarly, while the dipole moment of a charged system may influence physical interactions (e.g., torque in an external field), its value depends on the coordinate origin and thus lacks intrinsic meaning unless a reference frame is specified. Therefore, similar to $\vec{A}$ in the AB effect, the dipole moment of a charged molecule is *representation‐dependent* and should not be treated as a fundamental quantum observable. For a neutral system, the dipole moment is gauge‐invariant (i.e., origin‐independent) and thus a legitimate observable in Dirac's sense. For a charged system, however, the dipole moment is reference‐frame dependent and lacks this invariance, hence it fails to qualify as a fundamental quantum observable.

## 8 Good Practices for Reporting Dipole Moments of Charged Macromolecules

As just a couple of representative examples, and while not intending in any way to diminish the value of Creighton's [71] or van Holde's [72] books, certain statements regarding the dipole moments of some proteins are presented in these classic monographs ambiguously. For example, Creighton, on p. 340 states that “[*f*]*or horse cytochrome* c, *the charge distribution indicates a large dipole moment of just over 300 debye units, and the dipole axis passes through the presumed binding site. Consequently, protein interactions are not governed simply by diffusive encounters*” [71]. Neither the pH at which this dipole moment has been determined nor the total charge of cytochrome c is quoted in the text. If lack of specification in the book implies a neutral pH, and given a pI for this protein of ≈10.0, at pH = 7.0 cytochrome *c* will carry a net positive charge of +8 to +10 e depending on this protonation state and ionic strength. Belikova et al. estimate a net charge of +8 for ferric horse hearth cytochrome *c* at neutral pH [73]. In fact, cytochrome *c*, with its high isoelectric point and net positive charge at physiological pH, interacts favorably with the negatively charged phospholipid cardiolipin on the inner mitochondrial membrane [73, 74]. This electrostatic attractive interaction is important for anchoring cytochrome c close to Complex III (cytochrome bc_1_ complex) and Complex IV (cytochrome c oxidase), enabling efficient electron shuttling in the respiratory chain [74]. Thus, any discussion of this protein’s dipole moment must state the pH (especially if pH ≠ pI), the corresponding total charge, and the coordinate system used to evaluate the dipole.

The second example that we quote is from van Holde's [72] book. On page 156, the author provides a table (Table 6.3 of the book) where 4 proteins are listed along with their molecular weight and dipole moment magnitudes in D. The context suggests that the net charge of these proteins is zero, but no information is given regarding the pH, for example. The first listed protein, myoglobin, is charged (see our Table 1) yet it is listed in the book as having a dipole moment of 170 D. It is not clear whether this is the dipole moment at the pI of this protein or what coordinate system is used to define it in case it is not.

While we have chosen the geometric centroid of atomic positions as an origin due to its simplicity, there are other alternatives that can be more appropriate in other situations. For instance, the center of mass (COM) aligns with classical mechanics when evaluating rotational dynamics [75]. Alternatively, one can choose, say, the midpoint of the vector connecting the centers of positive and negative charge of the dipole moment. While none of these origins eliminate the origin‐dependence of the dipole moment in a charged system, they are chosen on purely practical bases.

It is, therefore, recommended to report the coordinate origin used (e.g., center‐of‐mass, geometric centroid, etc.) and specify unambiguously the orientations of the coordinate axes when discussing the dipole moments of a charged species. Dipole magnitudes of charged proteins can only be meaningfully compared after alignment of all compared structures to a common reference frame. When interpretability is paramount, consider calculating the dipole moments only for net‐neutral domains (or of the neutral overall protein, determined at its isoelectric point). Finally, the use of tools such as Dipole Moments Server (PDMS), GROMACS, PDB2PQR, or PyMOL scripts with explicit centering is recommended.

## 9 Conclusions

In charged proteins, the calculation and interpretation of dipole moments demand careful attention due to their dependence on the coordinate origin. This origin‐dependence arises because, for systems with net charge, the dipole moment vector transforms as $\vec{\mu^{\prime }}=\vec{\mu }-Q\vec{a}$, under a coordinate system displacement by $\vec{a}$. Consequently, the dipole moment of a charged system is not an intrinsic, origin‐independent property and cannot be regarded as a true physical observable unless the origin is fixed by external constraints.

Revisited theory and illustrative computational results demonstrate that origin‐induced changes in the dipole magnitude scale with both the total charge and the displacement vector and, in practice, with molecular size (e.g., radius of gyration) if the origin shift is of comparable magnitude. This implies that without a consistent choice of origin, comparative or functional interpretations may be misleading. We provide numerical estimates of these effects, which appear to be somewhat underrepresented in the current structural biology and bioinformatics literature.

From a quantum mechanical standpoint, the dipole operator is Hermitian (irrespective of the total charge of the molecule) and corresponds to a legitimate observable for neutral molecules for which its expectation value is invariant under origin shifts. However, for charged systems, this expectation value is gauge‐dependent, disqualifying it as an intrinsic property of the system. According to Dirac's transformation theory, only those quantities that remain invariant or transform in a simple well‐defined manner under coordinate changes qualify as observables. Hence, the dipole moment of a charged molecule is not an “observable”.

Although the approximation using fixed atom‐centered point charges lacks quantum mechanical detail (e.g., electron delocalization), it remains computationally tractable and widely employed in biomolecular simulations. This approach effectively folds electronic polarization into empirical charge distributions, providing a practical, if approximate, representation of molecular charge distribution. The resulting dipole is computed as a discrete sum over partial charges and Cartesian coordinates.

Despite the dependence of the dipole moment of a charged molecule on the coordinate system, the total electrostatic energy of a system remains invariant under origin shifts. Any apparent origin dependence in individual energy terms is an artifact that cancels out in the full expression of the potential and its associated field (Equations (1) and (2)) as required for an “observable”.

Finally, while the origin‐dependent dipole term contributes to the torque on a charged protein in an external electric field, it alone does not determine the orientation of the molecule. A complete and rigorous treatment must consider the entire charge distribution, not just its first moment. In the preface of the first edition of his book, Dirac's statement that: [64]

The important things in the world appear as the invariants (or more generally the nearly invariants, or quantities with simple transformation properties) of these transformations. The things we are immediately aware of are the relations of these nearly invariants to a certain frame of reference, usually one chosen so as to introduce special simplifying features which are unimportant from the point of view of general theory

encapsulates the central message of this paper: Only invariant or appropriately referenced quantities possess true physical meaning. Although well‐established, this point warrants reiteration in a biophysics context.

Before closing the paper, an open problem of fundamental interest is mentioned. A full quantum mechanical treatment, where the positions of the nuclei are represented by linear hermitian position operators, on par with the electronic coordinates, results in a rotationally symmetric Hamiltonian. Curie's principle stipulates that *the symmetries of the “causes” must be found in the “effects”, but the “effects” can be less symmetric than the “causes”* [76, 77]. Without symmetry‐breaking that lowers the symmetry of the effect, the latter must exhibit the symmetry of the cause. If the fully quantum non‐relativistic non‐Born‐Oppenheimer (BO) molecular Hamiltonian is the “cause”, then the “effect”, that is, the many‐particle molecular wavefunction, a product of the electronic and the nuclear wavefunctions, must be also rotationally‐symmetric. In this manner, the fully quantum solution of the Schrödinger equation cannot *even in principle* distinguish the isomers of, say, C_2_H_4_O_2_, namely, acetic acid, methyl formate, and oxiranol. A water molecule, for instance, would have a dipole moment of exactly zero since the distribution of the nuclei and those of the electrons are all rotationally‐symmetric. The introduction of the BO approximation breaks the symmetry by converting the operators for the nuclear positions into parameters and, hence, imposes “a structure” “by hand”. Only then does the permanent dipole moment of water emerge. For discussions of this problem, see the works of Woolley and Sutcliffe [78, 79, 80], and that of Lombardi, Fortin, and Martínez González [81, 82, 83, 84]. In the present paper, the BO approximation is a starting point and, hence, this problem does not occur.

## Conflicts of Interest

The authors declare no conflicts of interest.

## 10 Meaning and Implementation of Equations (5) and (6)

Equation (6), $\left|\Delta \vec{\mu }\right|=\left|Q\right|.|\vec{a}|$, follows directly from the origin‐shift relation ${\vec{\mu }}^{\prime }=\vec{\mu }-Q\;\vec{a}$ (Eq. (5)) and simply gives the magnitude of the translation term $\Delta \vec{\mu }\equiv {\vec{\mu }}^{\prime }-\vec{\mu }=-Q\;\vec{a}$. It does *not* by itself give the new dipole magnitude $\Delta |\vec{\mu }|\;=\;|{\vec{\mu }}^{\prime }|\;-\;|\vec{\mu }|$; since this depends on the angle *θ* between $\vec{\mu }$ and $\vec{a}$. Explicitly, with $\hat{r}=\;\vec{r}/|\vec{r}|$ and $\theta =∠\left(\vec{\mu },\vec{a}\right)$, we have:

(33) $$\begin{array}{c} |{\vec{\mu }}^{\prime }|=\sqrt{\;{\left|\vec{\mu }\right|}^{2}+{\left(Q\;a\right)}^{2}-2|\vec{\mu }\;|\;Q\;a\;\cos\theta \;} \end{array}$$

Thus Eq. (6) can be *somewhat deceptive* if read as implying a fixed proportionality between $|{\vec{\mu }}^{\prime }|$ (or $\Delta |\vec{\mu }|$) and ∣Q∣a. Such a proportionality would hold only if all compared proteins’ original dipoles $\vec{\mu }$ were *parallel and the origin is translated by the same* $\vec{a}$ (i.e., the same *θ* for all proteins), which is not the case for proteins listed in Table 1.

In general, $|\;{\vec{\mu }}^{\prime }|$ can either increase or decrease depending on cosθ (see the discussion around Equations (5)–(6) and Table 1). In Table 1, as a numerical example, we take a single displacement vector $\vec{a}=\left(+\;20,+\;20,+\;20\right)\;\overset{˚}{A}$, whose magnitude is $|\;\vec{a}|=34.64\;\overset{˚}{A}$. The new dipole moment magnitude $|{\vec{\mu }}^{\prime }|$ is obtained from the vector relation above (Equations (33)).

Conversely, knowing $|\;\vec{\mu }|$, $|\;{\vec{\mu }}^{\prime }|$, *Q*, and $|\;\vec{a}|$, one can solve for *θ*. The entries for myoglobin, for example, Q=+2, $|\vec{a}|=34.64\;\overset{˚}{A}$, $\begin{array}{c} |\;\Delta \vec{\mu }|=2\times 34.64\;e\cdot \overset{˚}{A}=69.28\;e\cdot \overset{˚}{A}\approx 332.8\;D\; \end{array}$, $|\;\vec{\mu }|=288\;D$, and $|\;{\vec{\mu }}^{\prime }|=469\;D$, the angle is obtained from:

(34) $$\begin{array}{c} \cos\theta =\frac{\;{\left|\vec{\mu }\right|}^{2}+{\left(Q\;a\right)}^{2}-{\left|{\vec{\mu }}^{\prime }\right|}^{2}\;}{\;2\;\left|\vec{\mu }\right|\;Q\;a\;}=\frac{288^{2}+332.8^{2}-469^{2}}{2\times 288\times 332.8}\\ \;\approx -0.137,\;\theta \approx 97.9^{\circ } \end{array}$$

The *θ* angles for all structures in Table 1 are: Lysozyme: *θ* ≈ 54.7^o^; myoglobin: *θ* ≈ 97.9^o^; histone H1.0: *θ* ≈ 41.7^o^; histone octamer: *θ* ≈ 138.7^o^; and RNase A: *θ* ≈ 8.6^o^.

In closing, one can verify the triangle inequality: $\begin{array}{c} |\left(\left|\vec{\mu }\right|-\left|Q\right|\;a\right)|\leq |{\vec{\mu }}^{\prime }|\leq |\vec{\mu }|+\left|Q\right|\;a \end{array}$, and that as a function of θ, the extrema depend on the sign of *Q*. If *Q* > 0, $|\;{\vec{\mu }}^{\prime }|$ is maximized at θ=π (anti‐parallel to $\vec{a}$) and minimized when θ=0, whereas if *Q* < 0 the situation is reversed (max at θ=0 and min at θ=π). All of that follows from Equations (5) and (6).

## Supporting information


**Data SI:** Supplementary Information.

## Data Availability

This is a theory paper, there are no data analyzed.

## Appendix A Methods

Protein structures were obtained from the Research Collaboratory for Structural Bioinformatics Protein Data Bank (RCSB PDB) [54]. Each downloaded PDB file was processed using PyMOL 3.1.0a0 Open‐Source (commit 9d3061ca58, released 2024‐09‐21) [49] to remove all non‐protein entities (e.g., ligands, water molecules, ions), ensuring compatibility with the AMBER force field used in subsequent steps. For homo‐multimeric proteins, monomeric chains were isolated, and the chain with the highest structural quality determined using the corresponding RCSB PDB structure validation report was selected for further analysis.

“Cleaned” monomeric PDB files were then processed using a locally installed version of PDB2PQR 3.7.1 [50, 51, 52, 53]. PDB2PQR was configured to apply the AMBER force field [43, 45, 46, 47] to assign atomic radii and assign partial charges. Structural protonation was carried out using PROPKA at the desired pH value. To generate titration curves and estimate both the net charge over a range of pH values and the isoelectric point (pI), PDB2PQR was executed iteratively from pH 0.0 to 14.0 in 0.1 pH unit increments. (All titration curve are available in the SI both graphically and numerically). The full procedure is detailed in the *titration_charge_profiler.py* script available in the project's GitHub repository [85].

Electric dipole moments were computed using the *calculate_absolute_dipole.py* script [85], which leverages PyMOL to calculate the electric dipole vector of each molecular structure without assuming a predefined coordinate origin. To assess the dependence of dipole calculations on origin placement, we used the *dipole_origin_dependence.py* script [85], which performs the following steps:

1. Loads the protein's PQR file into PyMOL.
2. Places two pseudoatoms: one at the protein's charge‐weighted center of geometry (PSDP) and one at the origin of the Cartesian coordinate system (PSDO).
3. Aligns PSDP to PSDO to translate the protein to the origin.
4. Removes the pseudoatoms and computes the dipole vector.
5. Repeats the process using a third pseudoatom placed at coordinates (20.0, 20.0, 20.0) Å (PSD20) in place of PSDO.
6. Records both resulting dipole vectors, their magnitudes, the difference vector (|Δ$\vec{\mu }$|), and the charge‐weighted radius of gyration.

All scripts and additional implementation details are available in the Supporting Information and through the GitHub repository linked therein [85].
