# Supplementary material for: Origin‐Dependence of Dipole Moments of Charged Proteins: Theoretical Foundations and Implications, Revisited
Source: J Comput Chem. 2025 Sep 25;46(25):e70207. doi: 10.1002/jcc.70207 (PMC12461687; doi:10.1002/jcc.70207)
Supplement: Supplementary file 1 — Data SI: Supplementary Information. [file JCC-46-0-s001.docx]

**Supporting Information for:**

Origin-Dependence of Dipole Moments of Charged Proteins:

Theoretical Foundations and Implications, Revisited

Islam K. Matar,^(a,b)^ Chérif F. Matta^(a,b)*^

(a) Department of Chemistry, Saint Mary's University, Halifax, NS, Canada, B3H 3C3.

(b) Department of Chemistry and Physics, Mount Saint Vincent University, Halifax, NS, Canada, B3M 2J6.

* E-mail: [cherif.matta@msvu.ca](mailto:cherif.matta@msvu.ca)

**Titration curves of proteins listed in Table 2**

Below are the titration curves for the proteins featured in Table 2 of the main article. These curves were generated using PROPKA (via PDB2PQR) and plotted with custom Python scripts. The y-axis reflects the total partial charge of the protein at varying pH, and the red dashed line indicates a specific target charge value used in dipole comparison.


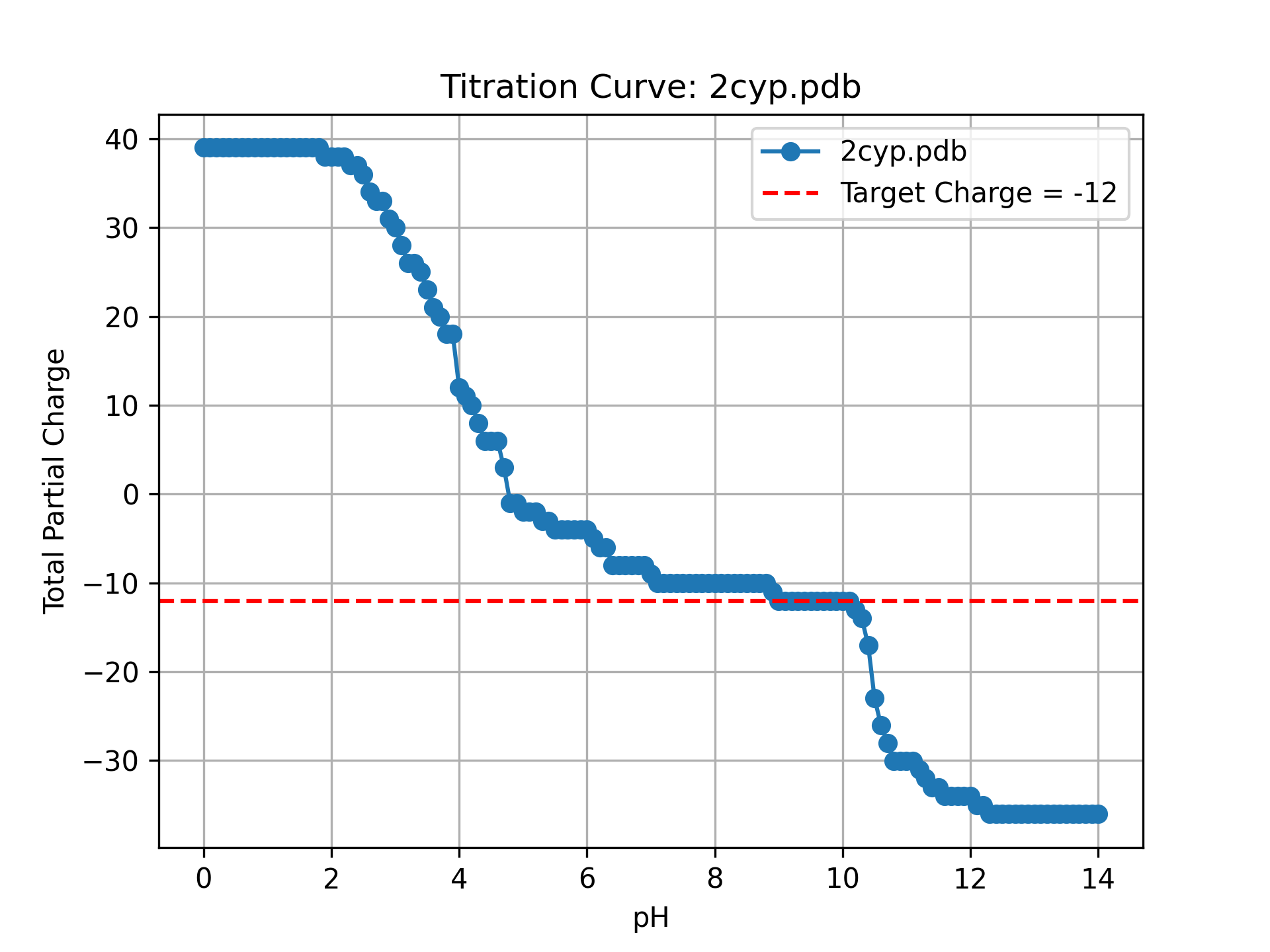


**Figure S1. Titration curve for Cytochrome c Peroxidase (PDB ID: 2CYP)**


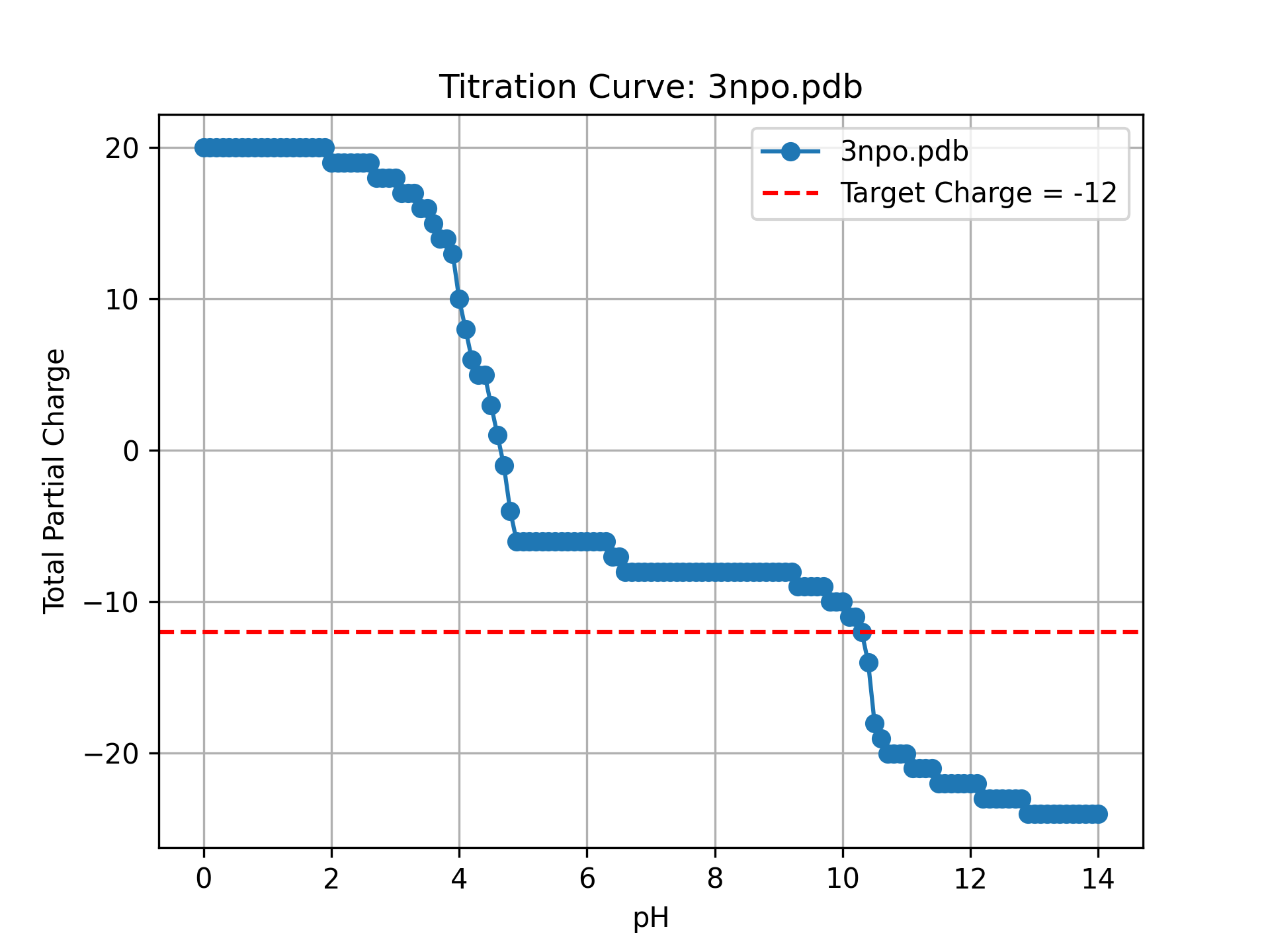


**Figure S2. Titration curve for β-Lactoglobulin (PDB ID: 3NPO)**


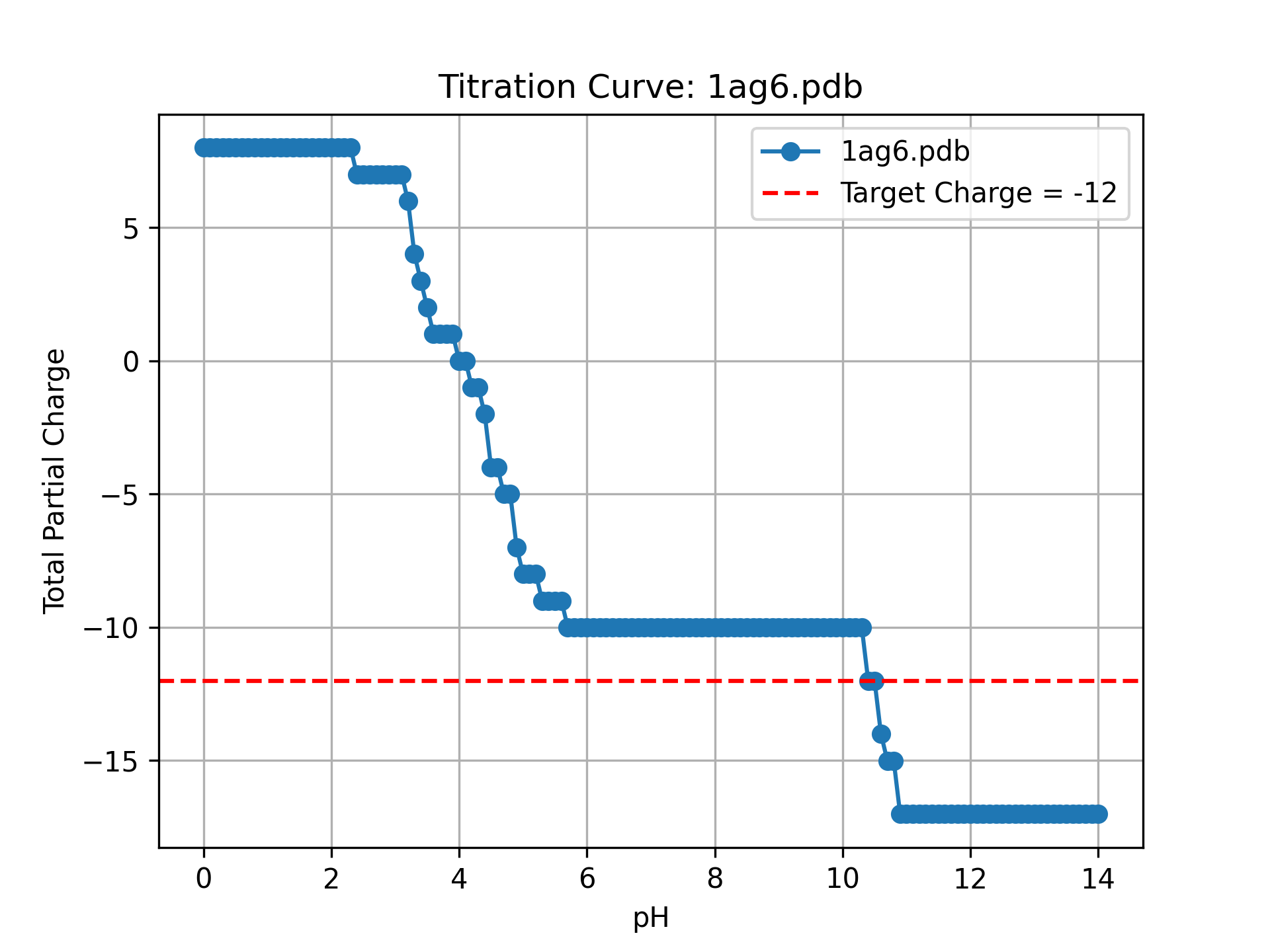


**Figure S3. Titration curve for Plastocyanin (PDB ID: 1AG6)**


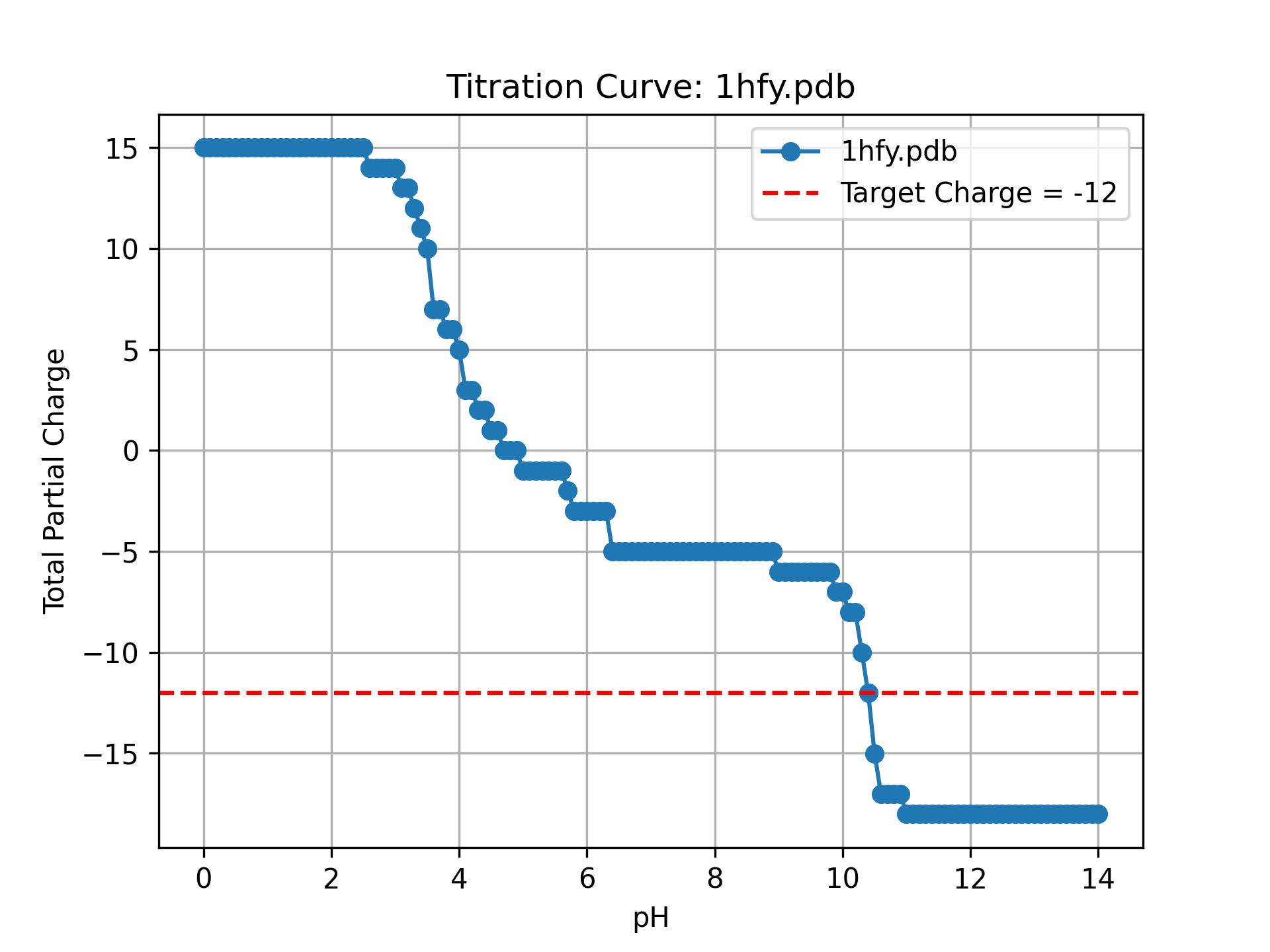


**Figure S4. Titration curve for α-Lactalbumin (PDB ID: 1HFY)**


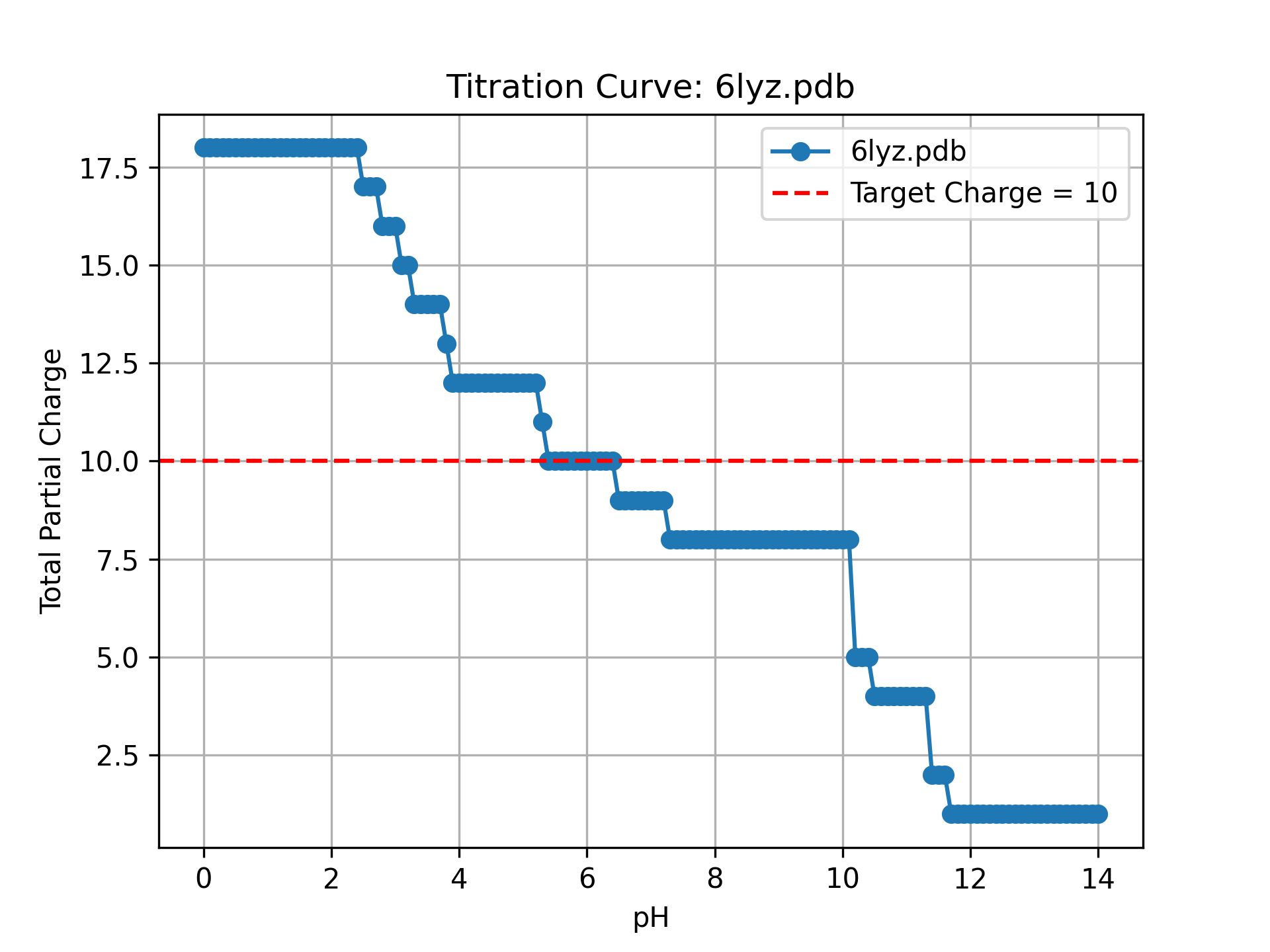


**Figure S5. Titration curve for Hen Egg-White Lysozyme (PDB ID: 6LYZ)**


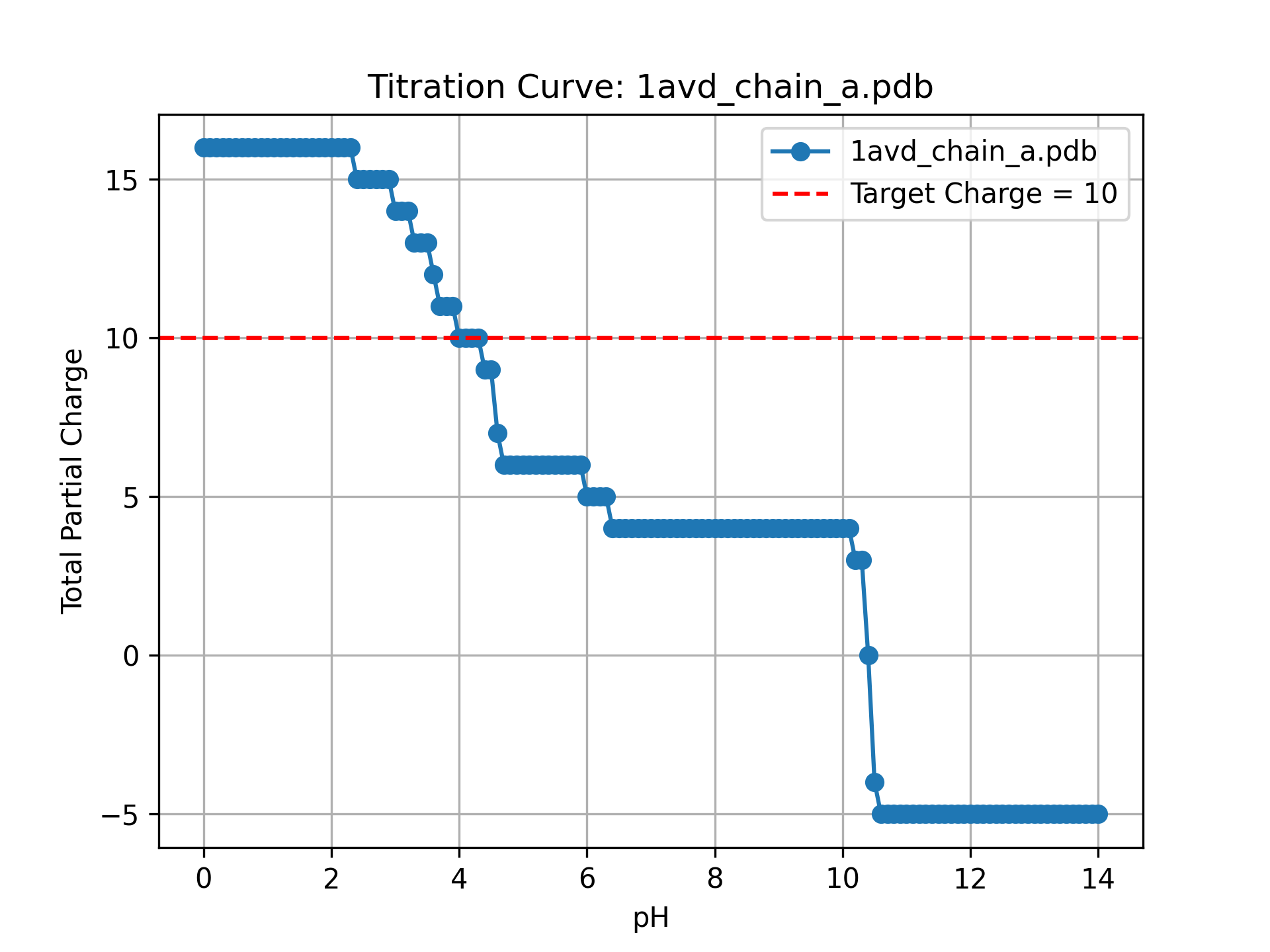


**Figure S6. Titration curve for Avidin (chain A, PDB ID: 1AVD)**


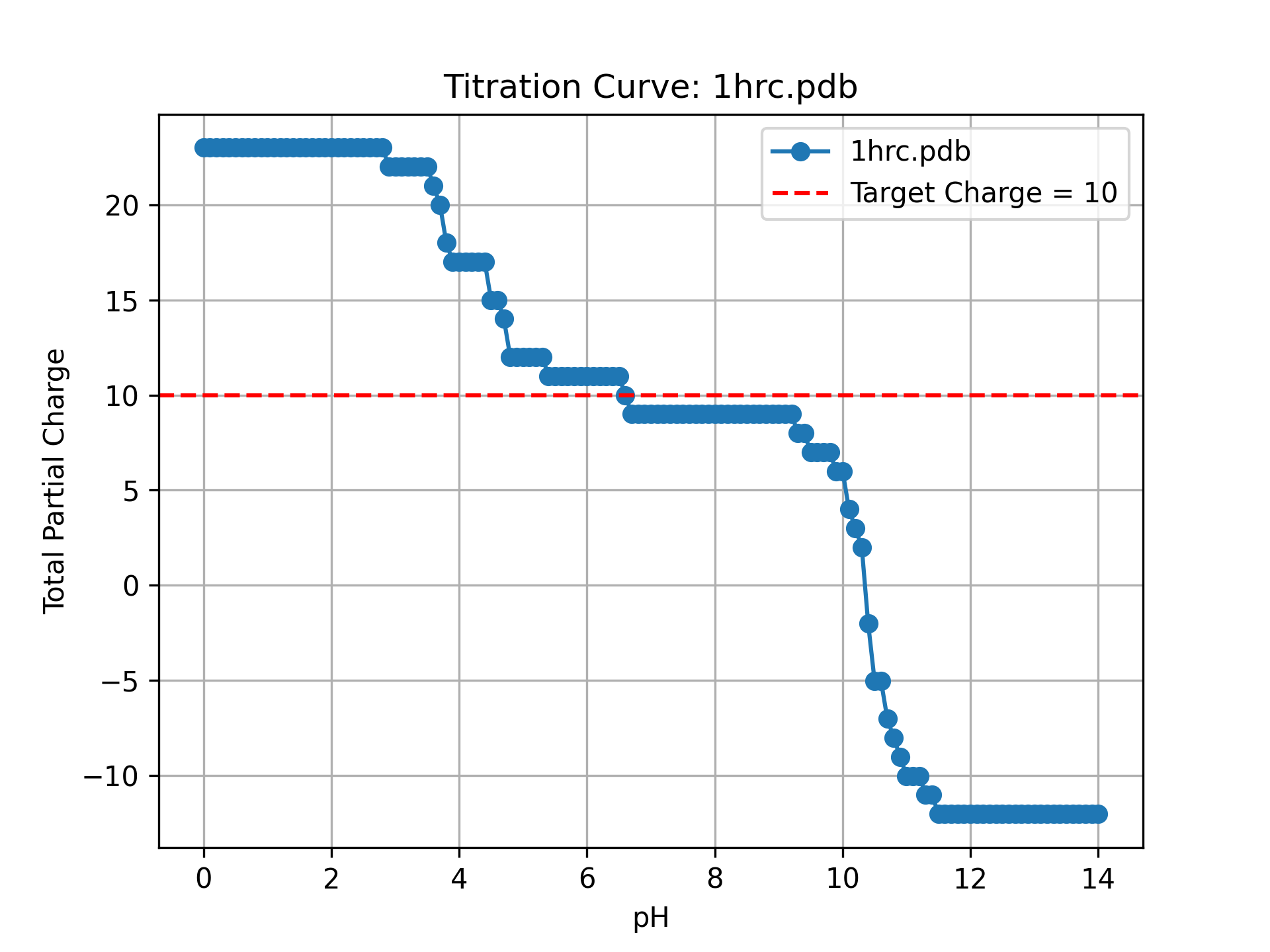


**Figure S7. Titration curve for Cytochrome c (PDB ID: 1HRC)**


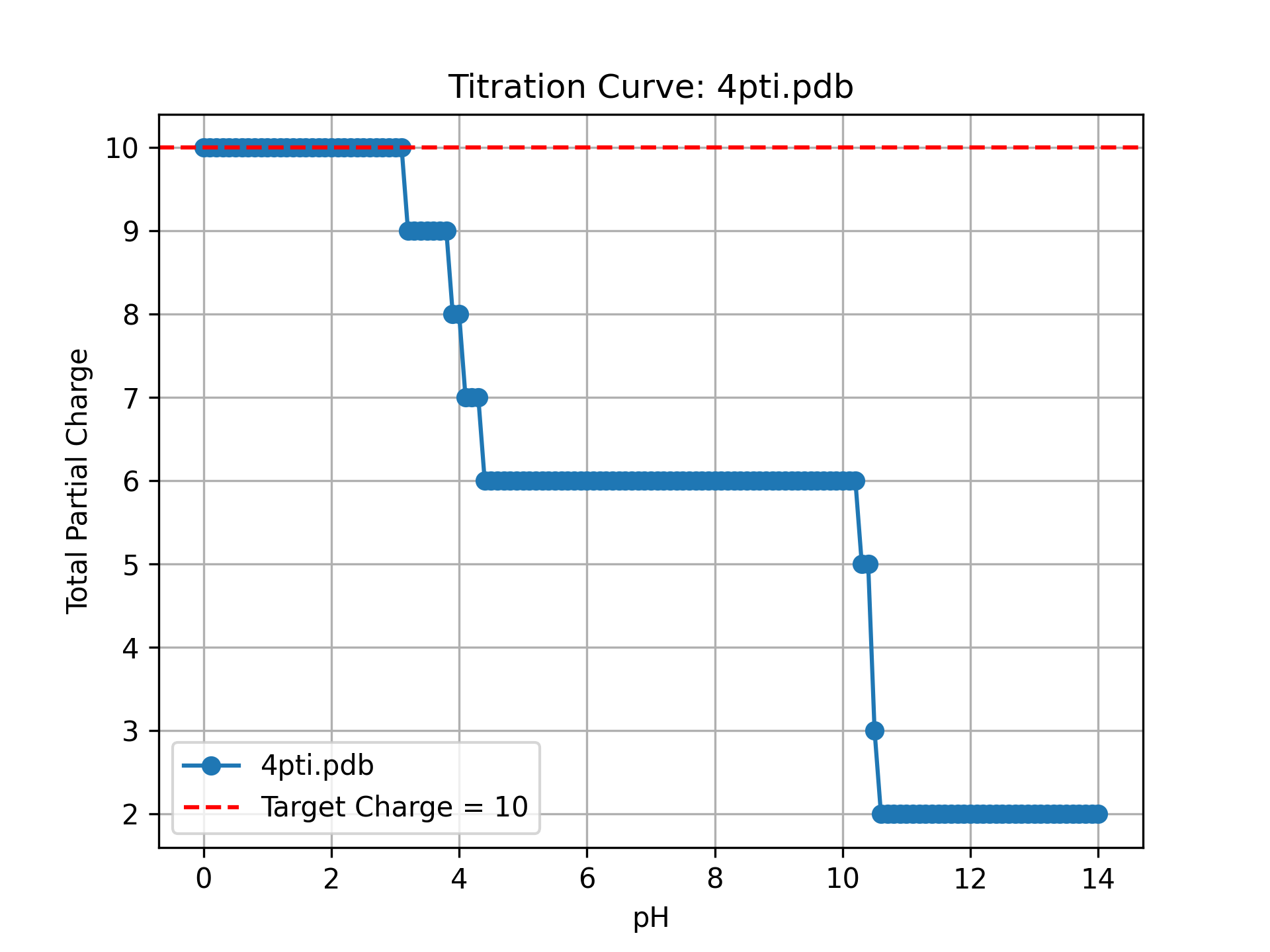


**Figure S8. Titration curve for Bovine Pancreatic Trypsin Inhibitor (PDB ID: 4PTI)**

**Numerical Values of Titration Curves (related to Table 2)**

**Table S1. Titration curves for positively charged proteins listing the respective charges in atomic units (in *e*). (Top part of Table 2).**

| **pH** | **1hrc.pdb** | **1avd_chain_a.pdb** | **4pti.pdb** | **6lyz.pdb** |
| --- | --- | --- | --- | --- |
| 0 | 23 | 16 | 10 | 18 |
| 0.1 | 23 | 16 | 10 | 18 |
| 0.2 | 23 | 16 | 10 | 18 |
| 0.3 | 23 | 16 | 10 | 18 |
| 0.4 | 23 | 16 | 10 | 18 |
| 0.5 | 23 | 16 | 10 | 18 |
| 0.6 | 23 | 16 | 10 | 18 |
| 0.7 | 23 | 16 | 10 | 18 |
| 0.8 | 23 | 16 | 10 | 18 |
| 0.9 | 23 | 16 | 10 | 18 |
| 1 | 23 | 16 | 10 | 18 |
| 1.1 | 23 | 16 | 10 | 18 |
| 1.2 | 23 | 16 | 10 | 18 |
| 1.3 | 23 | 16 | 10 | 18 |
| 1.4 | 23 | 16 | 10 | 18 |
| 1.5 | 23 | 16 | 10 | 18 |
| 1.6 | 23 | 16 | 10 | 18 |
| 1.7 | 23 | 16 | 10 | 18 |
| 1.8 | 23 | 16 | 10 | 18 |
| 1.9 | 23 | 16 | 10 | 18 |
| 2 | 23 | 16 | 10 | 18 |
| 2.1 | 23 | 16 | 10 | 18 |
| 2.2 | 23 | 16 | 10 | 18 |
| 2.3 | 23 | 16 | 10 | 18 |
| 2.4 | 23 | 15 | 10 | 18 |
| 2.5 | 23 | 15 | 10 | 17 |
| 2.6 | 23 | 15 | 10 | 17 |
| 2.7 | 23 | 15 | 10 | 17 |
| 2.8 | 23 | 15 | 10 | 16 |
| 2.9 | 22 | 15 | 10 | 16 |
| 3 | 22 | 14 | 10 | 16 |
| 3.1 | 22 | 14 | 10 | 15 |
| 3.2 | 22 | 14 | 9 | 15 |
| 3.3 | 22 | 13 | 9 | 14 |
| 3.4 | 22 | 13 | 9 | 14 |
| 3.5 | 22 | 13 | 9 | 14 |
| 3.6 | 21 | 12 | 9 | 14 |
| 3.7 | 20 | 11 | 9 | 14 |
| 3.8 | 18 | 11 | 9 | 13 |
| 3.9 | 17 | 11 | 8 | 12 |
| 4 | 17 | 10 | 8 | 12 |
| 4.1 | 17 | 10 | 7 | 12 |
| 4.2 | 17 | 10 | 7 | 12 |
| 4.3 | 17 | 10 | 7 | 12 |
| 4.4 | 17 | 9 | 6 | 12 |
| 4.5 | 15 | 9 | 6 | 12 |
| 4.6 | 15 | 7 | 6 | 12 |
| 4.7 | 14 | 6 | 6 | 12 |
| 4.8 | 12 | 6 | 6 | 12 |
| 4.9 | 12 | 6 | 6 | 12 |
| 5 | 12 | 6 | 6 | 12 |
| 5.1 | 12 | 6 | 6 | 12 |
| 5.2 | 12 | 6 | 6 | 12 |
| 5.3 | 12 | 6 | 6 | 11 |
| 5.4 | 11 | 6 | 6 | 10 |
| 5.5 | 11 | 6 | 6 | 10 |
| 5.6 | 11 | 6 | 6 | 10 |
| 5.7 | 11 | 6 | 6 | 10 |
| 5.8 | 11 | 6 | 6 | 10 |
| 5.9 | 11 | 6 | 6 | 10 |
| 6 | 11 | 5 | 6 | 10 |
| 6.1 | 11 | 5 | 6 | 10 |
| 6.2 | 11 | 5 | 6 | 10 |
| 6.3 | 11 | 5 | 6 | 10 |
| 6.4 | 11 | 4 | 6 | 10 |
| 6.5 | 11 | 4 | 6 | 9 |
| 6.6 | 10 | 4 | 6 | 9 |
| 6.7 | 9 | 4 | 6 | 9 |
| 6.8 | 9 | 4 | 6 | 9 |
| 6.9 | 9 | 4 | 6 | 9 |
| 7 | 9 | 4 | 6 | 9 |
| 7.1 | 9 | 4 | 6 | 9 |
| 7.2 | 9 | 4 | 6 | 9 |
| 7.3 | 9 | 4 | 6 | 8 |
| 7.4 | 9 | 4 | 6 | 8 |
| 7.5 | 9 | 4 | 6 | 8 |
| 7.6 | 9 | 4 | 6 | 8 |
| 7.7 | 9 | 4 | 6 | 8 |
| 7.8 | 9 | 4 | 6 | 8 |
| 7.9 | 9 | 4 | 6 | 8 |
| 8 | 9 | 4 | 6 | 8 |
| 8.1 | 9 | 4 | 6 | 8 |
| 8.2 | 9 | 4 | 6 | 8 |
| 8.3 | 9 | 4 | 6 | 8 |
| 8.4 | 9 | 4 | 6 | 8 |
| 8.5 | 9 | 4 | 6 | 8 |
| 8.6 | 9 | 4 | 6 | 8 |
| 8.7 | 9 | 4 | 6 | 8 |
| 8.8 | 9 | 4 | 6 | 8 |
| 8.9 | 9 | 4 | 6 | 8 |
| 9 | 9 | 4 | 6 | 8 |
| 9.1 | 9 | 4 | 6 | 8 |
| 9.2 | 9 | 4 | 6 | 8 |
| 9.3 | 8 | 4 | 6 | 8 |
| 9.4 | 8 | 4 | 6 | 8 |
| 9.5 | 7 | 4 | 6 | 8 |
| 9.6 | 7 | 4 | 6 | 8 |
| 9.7 | 7 | 4 | 6 | 8 |
| 9.8 | 7 | 4 | 6 | 8 |
| 9.9 | 6 | 4 | 6 | 8 |
| 10 | 6 | 4 | 6 | 8 |
| 10.1 | 4 | 4 | 6 | 8 |
| 10.2 | 3 | 3 | 6 | 5 |
| 10.3 | 2 | 3 | 5 | 5 |
| 10.4 | -2 | 0 | 5 | 5 |
| 10.5 | -5 | -4 | 3 | 4 |
| 10.6 | -5 | -5 | 2 | 4 |
| 10.7 | -7 | -5 | 2 | 4 |
| 10.8 | -8 | -5 | 2 | 4 |
| 10.9 | -9 | -5 | 2 | 4 |
| 11 | -10 | -5 | 2 | 4 |
| 11.1 | -10 | -5 | 2 | 4 |
| 11.2 | -10 | -5 | 2 | 4 |
| 11.3 | -11 | -5 | 2 | 4 |
| 11.4 | -11 | -5 | 2 | 2 |
| 11.5 | -12 | -5 | 2 | 2 |
| 11.6 | -12 | -5 | 2 | 2 |
| 11.7 | -12 | -5 | 2 | 1 |
| 11.8 | -12 | -5 | 2 | 1 |
| 11.9 | -12 | -5 | 2 | 1 |
| 12 | -12 | -5 | 2 | 1 |
| 12.1 | -12 | -5 | 2 | 1 |
| 12.2 | -12 | -5 | 2 | 1 |
| 12.3 | -12 | -5 | 2 | 1 |
| 12.4 | -12 | -5 | 2 | 1 |
| 12.5 | -12 | -5 | 2 | 1 |
| 12.6 | -12 | -5 | 2 | 1 |
| 12.7 | -12 | -5 | 2 | 1 |
| 12.8 | -12 | -5 | 2 | 1 |
| 12.9 | -12 | -5 | 2 | 1 |
| 13 | -12 | -5 | 2 | 1 |
| 13.1 | -12 | -5 | 2 | 1 |
| 13.2 | -12 | -5 | 2 | 1 |
| 13.3 | -12 | -5 | 2 | 1 |
| 13.4 | -12 | -5 | 2 | 1 |
| 13.5 | -12 | -5 | 2 | 1 |
| 13.6 | -12 | -5 | 2 | 1 |
| 13.7 | -12 | -5 | 2 | 1 |
| 13.8 | -12 | -5 | 2 | 1 |
| 13.9 | -12 | -5 | 2 | 1 |
| 14 | -12 | -5 | 2 | 1 |

**Table S2. Titration curves for negatively charged proteins listing the respective charges in atomic units (in *e*). (Bottom part of Table 2).**

| **pH** | **1hfy.pdb** | **1ag6.pdb** | **2cyp.pdb** | **3npo.pdb** |
| --- | --- | --- | --- | --- |
| 0 | 15 | 8 | 39 | 20 |
| 0.1 | 15 | 8 | 39 | 20 |
| 0.2 | 15 | 8 | 39 | 20 |
| 0.3 | 15 | 8 | 39 | 20 |
| 0.4 | 15 | 8 | 39 | 20 |
| 0.5 | 15 | 8 | 39 | 20 |
| 0.6 | 15 | 8 | 39 | 20 |
| 0.7 | 15 | 8 | 39 | 20 |
| 0.8 | 15 | 8 | 39 | 20 |
| 0.9 | 15 | 8 | 39 | 20 |
| 1 | 15 | 8 | 39 | 20 |
| 1.1 | 15 | 8 | 39 | 20 |
| 1.2 | 15 | 8 | 39 | 20 |
| 1.3 | 15 | 8 | 39 | 20 |
| 1.4 | 15 | 8 | 39 | 20 |
| 1.5 | 15 | 8 | 39 | 20 |
| 1.6 | 15 | 8 | 39 | 20 |
| 1.7 | 15 | 8 | 39 | 20 |
| 1.8 | 15 | 8 | 39 | 20 |
| 1.9 | 15 | 8 | 38 | 20 |
| 2 | 15 | 8 | 38 | 19 |
| 2.1 | 15 | 8 | 38 | 19 |
| 2.2 | 15 | 8 | 38 | 19 |
| 2.3 | 15 | 8 | 37 | 19 |
| 2.4 | 15 | 7 | 37 | 19 |
| 2.5 | 15 | 7 | 36 | 19 |
| 2.6 | 14 | 7 | 34 | 19 |
| 2.7 | 14 | 7 | 33 | 18 |
| 2.8 | 14 | 7 | 33 | 18 |
| 2.9 | 14 | 7 | 31 | 18 |
| 3 | 14 | 7 | 30 | 18 |
| 3.1 | 13 | 7 | 28 | 17 |
| 3.2 | 13 | 6 | 26 | 17 |
| 3.3 | 12 | 4 | 26 | 17 |
| 3.4 | 11 | 3 | 25 | 16 |
| 3.5 | 10 | 2 | 23 | 16 |
| 3.6 | 7 | 1 | 21 | 15 |
| 3.7 | 7 | 1 | 20 | 14 |
| 3.8 | 6 | 1 | 18 | 14 |
| 3.9 | 6 | 1 | 18 | 13 |
| 4 | 5 | 0 | 12 | 10 |
| 4.1 | 3 | 0 | 11 | 8 |
| 4.2 | 3 | -1 | 10 | 6 |
| 4.3 | 2 | -1 | 8 | 5 |
| 4.4 | 2 | -2 | 6 | 5 |
| 4.5 | 1 | -4 | 6 | 3 |
| 4.6 | 1 | -4 | 6 | 1 |
| 4.7 | 0 | -5 | 3 | -1 |
| 4.8 | 0 | -5 | -1 | -4 |
| 4.9 | 0 | -7 | -1 | -6 |
| 5 | -1 | -8 | -2 | -6 |
| 5.1 | -1 | -8 | -2 | -6 |
| 5.2 | -1 | -8 | -2 | -6 |
| 5.3 | -1 | -9 | -3 | -6 |
| 5.4 | -1 | -9 | -3 | -6 |
| 5.5 | -1 | -9 | -4 | -6 |
| 5.6 | -1 | -9 | -4 | -6 |
| 5.7 | -2 | -10 | -4 | -6 |
| 5.8 | -3 | -10 | -4 | -6 |
| 5.9 | -3 | -10 | -4 | -6 |
| 6 | -3 | -10 | -4 | -6 |
| 6.1 | -3 | -10 | -5 | -6 |
| 6.2 | -3 | -10 | -6 | -6 |
| 6.3 | -3 | -10 | -6 | -6 |
| 6.4 | -5 | -10 | -8 | -7 |
| 6.5 | -5 | -10 | -8 | -7 |
| 6.6 | -5 | -10 | -8 | -8 |
| 6.7 | -5 | -10 | -8 | -8 |
| 6.8 | -5 | -10 | -8 | -8 |
| 6.9 | -5 | -10 | -8 | -8 |
| 7 | -5 | -10 | -9 | -8 |
| 7.1 | -5 | -10 | -10 | -8 |
| 7.2 | -5 | -10 | -10 | -8 |
| 7.3 | -5 | -10 | -10 | -8 |
| 7.4 | -5 | -10 | -10 | -8 |
| 7.5 | -5 | -10 | -10 | -8 |
| 7.6 | -5 | -10 | -10 | -8 |
| 7.7 | -5 | -10 | -10 | -8 |
| 7.8 | -5 | -10 | -10 | -8 |
| 7.9 | -5 | -10 | -10 | -8 |
| 8 | -5 | -10 | -10 | -8 |
| 8.1 | -5 | -10 | -10 | -8 |
| 8.2 | -5 | -10 | -10 | -8 |
| 8.3 | -5 | -10 | -10 | -8 |
| 8.4 | -5 | -10 | -10 | -8 |
| 8.5 | -5 | -10 | -10 | -8 |
| 8.6 | -5 | -10 | -10 | -8 |
| 8.7 | -5 | -10 | -10 | -8 |
| 8.8 | -5 | -10 | -10 | -8 |
| 8.9 | -5 | -10 | -11 | -8 |
| 9 | -6 | -10 | -12 | -8 |
| 9.1 | -6 | -10 | -12 | -8 |
| 9.2 | -6 | -10 | -12 | -8 |
| 9.3 | -6 | -10 | -12 | -9 |
| 9.4 | -6 | -10 | -12 | -9 |
| 9.5 | -6 | -10 | -12 | -9 |
| 9.6 | -6 | -10 | -12 | -9 |
| 9.7 | -6 | -10 | -12 | -9 |
| 9.8 | -6 | -10 | -12 | -10 |
| 9.9 | -7 | -10 | -12 | -10 |
| 10 | -7 | -10 | -12 | -10 |
| 10.1 | -8 | -10 | -12 | -11 |
| 10.2 | -8 | -10 | -13 | -11 |
| 10.3 | -10 | -10 | -14 | -12 |
| 10.4 | -12 | -12 | -17 | -14 |
| 10.5 | -15 | -12 | -23 | -18 |
| 10.6 | -17 | -14 | -26 | -19 |
| 10.7 | -17 | -15 | -28 | -20 |
| 10.8 | -17 | -15 | -30 | -20 |
| 10.9 | -17 | -17 | -30 | -20 |
| 11 | -18 | -17 | -30 | -20 |
| 11.1 | -18 | -17 | -30 | -21 |
| 11.2 | -18 | -17 | -31 | -21 |
| 11.3 | -18 | -17 | -32 | -21 |
| 11.4 | -18 | -17 | -33 | -21 |
| 11.5 | -18 | -17 | -33 | -22 |
| 11.6 | -18 | -17 | -34 | -22 |
| 11.7 | -18 | -17 | -34 | -22 |
| 11.8 | -18 | -17 | -34 | -22 |
| 11.9 | -18 | -17 | -34 | -22 |
| 12 | -18 | -17 | -34 | -22 |
| 12.1 | -18 | -17 | -35 | -22 |
| 12.2 | -18 | -17 | -35 | -23 |
| 12.3 | -18 | -17 | -36 | -23 |
| 12.4 | -18 | -17 | -36 | -23 |
| 12.5 | -18 | -17 | -36 | -23 |
| 12.6 | -18 | -17 | -36 | -23 |
| 12.7 | -18 | -17 | -36 | -23 |
| 12.8 | -18 | -17 | -36 | -23 |
| 12.9 | -18 | -17 | -36 | -24 |
| 13 | -18 | -17 | -36 | -24 |
| 13.1 | -18 | -17 | -36 | -24 |
| 13.2 | -18 | -17 | -36 | -24 |
| 13.3 | -18 | -17 | -36 | -24 |
| 13.4 | -18 | -17 | -36 | -24 |
| 13.5 | -18 | -17 | -36 | -24 |
| 13.6 | -18 | -17 | -36 | -24 |
| 13.7 | -18 | -17 | -36 | -24 |
| 13.8 | -18 | -17 | -36 | -24 |
| 13.9 | -18 | -17 | -36 | -24 |
| 14 | -18 | -17 | -36 | -24 |

**Source Code and Scripts**

The full set of Python and PyMOL scripts used to process protein structures, compute dipole moments, apply coordinate transformations, and generate titration plots are available on GitHub:

<https://github.com/Orbitaliant/CODDM-CPs>

This repository includes:
- Dipole calculation modules using atomic coordinates and partial charges.
- pH-dependent charge assignment workflow using PDB2PQR and PROPKA.
- Titration curve plotting utilities and visualization scripts.
- Documentation on usage and dependencies.

**Data Processing Notes**

- All input PDB files were preprocessed using PyMOL and then PDB2PQR with the AMBER force field.

- Coordinate origin displacements used in simulations were typically (+20, +20, +20) Å.
- All dipole moments were calculated in Debye (D), with unit conversions noted in the main text.
